# Adverse Physical Health Outcomes and Healthcare Service Utilization in Siblings of Children With Cancer: A Systematic Review

**DOI:** 10.1002/cam4.71035

**Published:** 2025-07-25

**Authors:** Victorine Sirveaux, Lily Puterman‐Salzman, Yue Qian Zhang, Eleni Sotirakos, Philippe Dodin, Guillaume Dumas, Eyal Cohen, Nadia Roumeliotis, Petros Pechlivanoglou, Hallie Coltin

**Affiliations:** ^1^ Centre de Recherche Azrieli du CHU Sainte‐Justine Montréal Quebec Canada; ^2^ Université de Rennes Rennes France; ^3^ Faculty of Medicine Université de Montréal Montréal Quebec Canada; ^4^ SickKids Research Institute Toronto Ontario Canada; ^5^ CHU Sainte‐Justine Montréal Quebec Canada; ^6^ Division of Paediatric Medicine, Department of Paediatrics, the Hospital for Sick Children University of Toronto Toronto Ontario Canada; ^7^ Division of Critical Care, Department of Pediatrics, CHU Sainte‐Justine Université de Montréal Montréal Quebec Canada; ^8^ Division of Hematology‐Oncology, Department of Pediatrics, CHU Sainte Justine Université de Montréal Montréal Quebec Canada

**Keywords:** adverse physical health, healthcare service utilization, pediatric cancer, siblings

## Abstract

**Introduction:**

Siblings of children with cancer may be vulnerable to compromised long‐term health. We aimed to describe the frequency (prevalence, incidence) of adverse physical health outcomes and healthcare service utilization among siblings of children with cancer and compare the risk of the above outcomes to siblings of children without cancer.

**Methods:**

We searched Ovid MEDLINE, Embase, Cochrane Central Register of Controlled Trials, CINAHL, and Clarivate Web of Science through June 15, 2024. We included English and French‐language studies, both with and without a healthy control population, that reported adverse physical health outcomes and/or healthcare service utilization outcomes among siblings of children with cancer. Studies focusing exclusively on mental health or quality of life were excluded. Abstracts were screened by two reviewers; full‐text articles underwent data abstraction and risk of bias assessment. Results were synthesized descriptively.

**Results:**

Of 26,570 studies screened, 44 were included. Heterogeneity was observed in all reported outcomes: mortality; cancer; organ system disease; overweight/obesity; pain; congenital anomalies; comorbidities; infections; amputations; adverse health behavior (smoking, alcohol consumption); infertility; healthcare service utilization (hospitalization, emergency department/urgent care visits, prescriptions). We detected a trend toward increased risk of cancer, hospitalizations, and prescription medication use compared to control siblings. Significant study heterogeneity rendered meta‐analyses inappropriate.

**Conclusions:**

Siblings of children with cancer are likely vulnerable to various adverse health outcomes. However, the published literature is widely heterogeneous regarding study design, populations, and outcomes measurements, limiting our comprehensive analysis of risk. Future research with homogenized methodology is needed to better quantify risk, which would inform targeted surveillance guidelines and interventions.

## 1 Introduction

### 1.1 Rationale

A childhood cancer diagnosis is a life‐altering event for the entire family. Siblings of children with cancer are vulnerable to chronic stress [1], adverse physical responses such as pain and sleep impairment [2], and may be at increased risk of compromised long‐term health and disproportionate healthcare utilization. Siblings of children with diverse chronic conditions have been reported to have an increased risk of adverse physical health diagnoses, healthcare service utilization, and prescription medication use [3, 4]. Given the life‐threatening nature of a pediatric cancer diagnosis and the lengthy, intense treatment required for cure, siblings of children with cancer may also be at significant risk of adverse health outcomes. However, whether siblings of children with cancer experience disproportionately adverse health outcomes remains poorly understood.

### 1.2 Objectives

Extrapolating from the aforementioned work focused on siblings of children with chronic disease, we hypothesized that siblings of children with cancer are at an increased risk of adverse physical health outcomes and healthcare service utilization compared to siblings of children without cancer. We therefore conducted a systematic review of observational studies evaluating the frequency of adverse physical health conditions and healthcare service utilization among siblings of children with cancer, comparing their risks to siblings of healthy children when possible.

## 2 Methods

We undertook a systematic review and narrative synthesis evaluating the association between a child's cancer diagnosis and their sibling's physical health and healthcare service utilization. The protocol was established in accordance with the Preferred Reporting Items for Systematic Review and Meta‐Analysis guidelines [5] (Supplement) and was registered in the International Prospective Register of Systematic Reviews (CRD42023440951).

### 2.1 Search Strategy and Data Items

With the aid of a library scientist (PD), we searched Ovid MEDLINE, Embase, Cochrane Central Register of Controlled Trials, CINAHL, and Clarivate Web of Science through June 15, 2024 (search terms in Table S1). We identified additional studies by snowballing. We included English‐ and French‐language observational studies, both with and without healthy control groups, that reported adverse physical health outcomes and/or healthcare service utilization among siblings of children with cancer. Specifically, outcomes included mortality, cancer, organ system‐based adverse physical health outcomes, overweight/obesity, pain, congenital anomalies, comorbidities, infections, adverse health behaviors, and healthcare service utilization (Table 1).

**TABLE 1:** List of included outcomes.

Categories	Included outcomes
**Adverse physical health outcomes**	
Mortality	All‐cause mortality, cancer‐related mortality
Cancer	SMN, history of cancer, history of solid tumors
**Organ system‐based adverse physical health outcomes**	
Neurological	Neurological conditions or impairments, hospitalization for nervous system conditions, neurocognitive deficits, sensory neuropathy, hypoesthesia, balance disorders, dysphagia or chewing difficulties, anosmia or ageusia, speech disorders, epilepsy
Cardiac and cerebrovascular	Cardiac, cerebrovascular, and cardiopulmonary conditions or impairments, hospitalization for circulatory disease, cardiac disease, ischemic heart disease, CHF and cardiomyopathy, cardiovascular risk factors, hypertension, blood pressure and heart medications, arrhythmia, stroke
Ophthalmologic	Eye and visual conditions or impairments, hospitalization for eye and mastoid conditions, cataracts, eye movement disorders, dry eye syndrome
Auditory	Ear conditions or impairment, hearing loss, tinnitus
Endocrine	Endocrine conditions or impairments, hospitalization for endocrine disease, hyper‐ or hypothyroidism, thyroid nodules or tumor, diabetes
Pulmonary	Any pulmonary conditions or impairments, hospitalization for respiratory conditions, asthma, dyspnea on exertion, drugs for respiratory disease, lung fibrosis, chronic cough, chest wall abnormalities
Bone/musculoskeletal	Musculoskeletal conditions or impairments, hospitalization for musculoskeletal conditions, osteoporosis and osteopenia, major joint replacement, scoliosis
Renal	Renal and urinary tract conditions or impairments, renal failure, repeated cystitis, repeated nephritis
Hepatic	Hepatic conditions or impairments
Genitourinary	Genitourinary conditions or impairments, hospitalization for genitourinary conditions
Digestive/gastrointestinal	Digestive/gastrointestinal conditions, hospitalization for digestive conditions, chronic constipation or diarrhea, gastro‐esophageal reflux disease, problems with esophagus, frequent nausea
Overweight/obesity	Overweight, obesity
Pain and other symptoms	Pain, abnormal sensations, fatigue syndromes and chronic fatigue, headaches and migraines, stomach ache, use of prescribed pain medications, prolonged pain in arms, legs, back, prolonged pain in bones and joints
Amputations	Limb amputations
Infertility	Decreased fertility, consultation with reproductive specialist, ART
Congenital anomalies	Congenital anomalies/birth defects
Comorbidities	Comorbidities, any chronic CHC, clinically relevant validated outcomes (2 to 4)
Infections	HCV positivity
Adverse health behaviors	Smoking, excessive alcohol consumption
**Healthcare service utilization**	
Healthcare service utilization	Hospitalization, ED/urgent care visits, prescriptions

Abbreviations: ART, assisted reproductive treatment; CHC, chronic health conditions; CHF, congestive heart failure; ED, emergency department; HCV, hepatitis C virus; SMN, second malignant neoplasm.

### 2.2 Eligibility Criteria

We excluded studies which focused exclusively on mental health or quality of life measures, review articles, gray literature, studies which only included children with cancer and/or extended family members (e.g., grandparents), and if the reported health outcomes were not clinical diagnoses (e.g., nondiagnostic bloodwork, continuous anthropometric measurements).

### 2.3 Selection Process and Data Collection

All identified references were transferred into Covidence [6]. Duplicated studies were removed before screening. Two reviewers (LPS, ES) screened titles and abstracts, followed by full‐text articles. Authors were contacted up to two times when data were incomplete or unclear. Data from included studies were reviewed and extracted using a predetermined data extraction tool (Table S2). Sample extractions were tested and validated by HC. Two reviewers (LPS, VS) extracted data independently.

### 2.4 Study Risk of Bias Assessment

Two reviewers (LPS, VS) assessed the risk of bias using the Joanna Briggs Institute (JBI) checklist for prevalence studies [7]. Accordingly, studies scoring 3 or lower were defined as low risk, 4–5 as moderate risk, and 5–6 as high risk of bias. Discrepancy was reviewed and resolved by consensus by HC. In case of participants' overlap between studies (e.g., Childhood Cancer Survivor Study (CCSS) publications), the largest and most recent study was included in the analysis for each outcome. We used a narrative review to summarize and report results by outcome type. Outcomes with two or more measures of risk were summarized in a forest plot using the software RStudio [8]. Results reporting a 95% confidence interval (CI) that did not cross the neutral value 1 were considered statistically significant.

## 3 Results

A total of 51,026 records were identified in the literature search, and 357 studies underwent full‐text review. Forty‐four studies were included in our review (Figure S1) and a total of 84,637 siblings and 102,377 controls were included (Table 2). The studies spanned 12 countries on three continents. All included studies were retrospective, with the exception of one prospective study [9]. Three (7%) studies were retrospective cohort studies [12, 49, 51], four (9%) were case–controls [9, 10, 11, 12], and 37 (84%) were population‐based registry studies [10, 11, 13, 14, 15, 16, 17, 18, 19, 20, 21, 22, 23, 24, 25, 26, 27, 28, 29, 30, 31, 32, 33, 34, 35, 36, 37, 38, 39, 40, 41, 42, 43, 44, 45, 46, 47]. Thirty‐four (77%) were questionnaire/survey/interview‐based [9, 10, 11, 12, 14, 15, 16, 17, 18, 19, 20, 21, 24, 25, 26, 27, 28, 29, 30, 32, 33, 34, 35, 36, 37, 38, 39, 40, 42, 43, 44, 45, 46, 48] whereas the remaining studies reported medical record and/or insurance data. The results summarized by outcomes are presented in Table S3.

**TABLE 2:** Characteristics of included studies.

Source	Study design	Period of reference^a^	Pathology of the index child	Source of data	No. of siblings enrolled	Age of siblings (years)	Outcomes	Control group
Armstrong et al., USA, Canada, 2013	Population‐based^b^ cohort	1970–1986	All childhood cancer	Self‐report	3159	Median [range]: 36.0 [7.1–62.6]	Cardiovascular risk factors	No
Auger et al., Canada, 2022	Population‐based^c^ cohort	2006–2019	All childhood cancer	ICD‐3, hospital discharge	1600	NA	Hospitalization	Yes
Baker et al., USA, 2010	Retrospective cohort	1974–1998	ALL and AML treated with hematopoietic cell transplantation	Diagnosis by healthcare provider	319	Median: 44	Organ system impairments	No
Belle et al., Switzerland, 2018	Population‐based^d^ cohort	2007–2013	Leukemia	Self‐reported BMI	819	Range [15–45]	Overweight	Yes
Belle et al., Switzerland, 2018	Population‐based^d^ cohort	1976–2005	ALL, NHL, and HL	Self‐report	564	NA	Overweight	Yes
Birch et al., England, 1990	Population‐based^e^ cohort	1954–1987	STS (including RMS, fibrosarcoma and fibrous histiocytoma)	ICD‐O, Self‐report	411	Median: 22	Cancer	No
Bouwman et al., the Netherlands, 2024	Population‐based^f^, cross‐sectional	1963–2001	All childhood cancer	Self‐report, ICD‐3	906	Mean [SD]: 33.7 [9.8]	Overweight, obesity, smoking, limb amputation, comorbidities	Yes
Bowers et al., USA, Canada, 2005	Population‐based^b^ cohort	1970–1986	Pediatric HL	Self‐report	3846	Mean [SD]: 28.8 [9.3]	Strokes	No
Buchbinder et al., USA, Canada, 2015	Population‐based^b^ cohort	1970–1986	CCSS‐specific cancer^h^	Self‐report	1974	Median [range]: 38 [31–44]	Tobacco use	Yes
Byrne et al., USA, 1995	Population‐based^g^ cohort	1946–1962	Retinoblastoma	Self‐report	84	NA	Cancer, birth defects	No
Chow et al., USA, Canada, 2014	Population‐based^b^ cohort	1970–1986	CCSS‐specific cancer^h^	Self‐report, ICD‐9, ICD‐10	4023	Median [range]: 34 [3–63]	Congestive heart failure	No
Chow et al., USA, Canada, 2017	Population‐based^b^ cohort	1970–1986	Childhood ALL	Self‐report, ICD‐9, ICD‐10	4.023	NA	Ischemic heart disease, stroke	No
Claessens et al., the Netherlands, 2024	Population‐based^f^, cross‐sectional	1963–2001	All childhood cancer	Self‐report	185	NA	Reproductive health	No
Del Risco Kollerud et al., Norway, 2018	Population‐based^i^ cohort	1960–2001	All solid childhood tumors, except lymphomas	ICD‐7, ICD‐8, Medical Birth Registry of Norway	NA	Mean [SD]: 26 [12]	Cancer	No
Desai et al., Canada, 2021	Population‐based^j^ cohort	1988–2016	All childhood cancer	Health administrative data	7591	Median [IQR]: 5 [0–10]	CHC, healthcare use	Yes
Dixon et al., USA, Canada, 2020	Population‐based^b^ cohort	1970–1999	Childhood ALL	Self‐report	5051	Median [range]: 35.9 [3.1–68.9]	CHC	No
Dixon et al., USA, Canada, 2022	Population‐based^b^ cohort	1970–1999	Childhood ALL	Self‐report	4693	Median [range]: 36.7 [18.0–68.9]	Health status	No
Ehrhardt et al., USA, Canada, 2019	Population‐based^b^ cohort	1970–1999	NHL treated with the LMB regimen	Self‐report	1029	Median [range]: 27.9 [0.3–52.3]	CHC	No
Essig et al., USA, Canada, 2014	Population‐based^b^ cohort	1970–1986	Childhood ALL	Self‐report	2232	NA	CHC	No
Feudtner et al., USA, 2021	Retrospective cohort	2015–2016	All childhood cancer	ICD‐10‐CM, health insurance claims	2072	Mean [SD]: 12.1 [6.5]	Healthcare encounters, diagnoses, prescriptions	Yes
Friedman et al., USA, Canada, 2005	Population‐based^b^ cohort	1970–1986	CCSS‐specific cancer^h^	Self‐report, oncologist review	26,193	Mean [SD]: 26.6 [10.2]	Cancer	Yes
Hartley et al., UK, 1991	Population‐based^e^ cohort	1954–1988	Ewing's tumor	Self‐report, records and registrations	124	Median: 27	Cancer	No
Hayek et al., USA, Canada, 2020	Population‐based^b^ cohort	1970–199	CCSS‐specific cancer^h^	Self‐report	2097	Mean [SD]: 24.5 [8.4]	Frailty	No
Infante‐Rivard et al., Canada, 2001	Population‐based^k^ case–control	1980–1993	Childhood ALL	Self‐report, ICD‐9	491	Mean: 5.0; Median [SD]: 3.96 [3.7]; IQR: 3.98	Congenital anomalies	Yes
Infante‐Rivard et al., Canada, 2003	Population‐based^k^ case–control	1980–1998	Childhood ALL	Self‐report, ICD‐9	792	NA	Hematopoietic malignancies	Yes
Landy et al., USA, Canada, 2013	Population‐based^b^ cohort	1999–2003	CCSS‐specific cancer^h^	Hospital visits, body fat measures	30	NA	Overweight, obesity	No
Lown et al., USA, Canada, 2012	Population‐based^b^ cohort	1970–1986	All childhood cancer	Self‐report	3034	Median [range]: 29 [18–56]	Risky and heavy drinking	Yes
Lu et al., USA, Canada, 2011	Population‐based^b^ cohort	1970–1986	CCSS‐specific cancer^h^	Self‐report	3034	NA	Pain conditions & prescriptions	No
Meacham et al., USA, Canada, 2009	Population‐based^b^ cohort	1970–1986	CCSS‐specific cancer^h^	Self‐report	2936	Mean [range]: 33.4 [9.6–58.4]	Diabetes mellitus	No
Molgaard‐Hansen et al., Denmark, Finland, Iceland, Norway, Sweden, 2009	Population‐based^l^ cohort	1984–2003	Childhood AML	Self‐report	86	NA	Healthcare encounters	No
Möttönen et al., Finland, 1995	Prospective case–control	1987–1989	Childhood ALL	Self‐report	18	Mean [range]: 10.6 [2.8–23.2]	Headache, stomach ache	Yes
Ng et al., USA, 2005	Cross‐sectional	1969–1996	Pediatric HL	FACIT‐F, self‐report	224	Median: 46	Fatigue	No
Oeffinger et al., USA, Canada, 2003	Population‐based^b^ cohort	1970–1986	Childhood ALL	Self‐reported BMI	2516	Mean [SD]: 29.0 [7.3]	Overweight, obesity	No
Oeffinger et al., USA, Canada, 2006	Population‐based^b^ cohort	1970–1986	CCSS‐specific cancer^h^	Self‐report	3034	Mean [range]: 29.2 [18.0–56.0]	CHC	No
Ou et al., USA, 2017	Retrospective cohort	1998–2013	Childhood ALL	EDW, ICD‐9	2032	Mean [range]: 11.3 [5.1–24.3]	Hospitalization	Yes
Penson et al., the Netherlands, 2023	Population‐based^f^ cross‐sectional	1963–2001	All childhood cancer	Self‐report	449	Median [SD]: 36.8 [10.2]	Chronic fatigue	No
Rueegg et al., Switzerland, 2012	Population‐based^d^ cohort	1976–2003	All childhood cancer	Self‐report	534	NA	Medical conditions limiting sports	No
Sherief et al., Egypt, 2019	Cross‐sectional	2016–2018	All childhood cancer	Blood sample, PCR	274	NA	HCV infection	No
Sláma et al., Switzerland, 2024	Population‐based^d^ cohort	1976–2015	Childhood LCH	Self‐report	999	Median [IQR]: 25 [18–32]	CHC	No
Streefkerk et al., the Netherlands, 2024	Population‐based^f^ cohort	1963–2001	All childhood cancer	Self‐report	1066	Median [range]: 31.9 [24.5–39.4]	Organ system impairments	No
Tacyildiz et al., Turkey, 2024	Retrospective case–control	1998–2019	Pediatric HL	Self‐report, review of patient charts	56	Median: 19.81 ± 5.85	Organ system disease, health care utilization	No
Van der Plas et al., USA, Canada, 2024	Population‐based^b^ cross‐sectional	1970–1999	Pediatric rhabdomyosarcoma	Self‐report	706	Median [IQR]: 32 [27–38]	Pain, health perceptions	No
Winther et al., Denmark, Finland, Iceland, Norway, Sweden, 2001	Population‐based^m^ cohort	1943–1993	All childhood cancer	ICD‐7	42,277	NA	Cancer	No
Yu et al., Denmark, Sweden, 2017	Population‐based^n^ cohort	1973–2008	All childhood cancer	Death registers, ICD‐8, ICD‐9, ICD‐10	NA	Median [IQR]: 7.0 [3.3–12.1]	Mortality	Yes

Abbreviations: ALL, acute lymphoblastic leukemia; AML, acute myeloid leukemia; BMI, Body mass index; CCSS, Childhood cancer survivor study; CHC, chronic health conditions; CNS, central nervous system; EDW, enterprise data warehouse (electronic medical records); FACIT‐F, Functional assessment of chronic illness therapy – Fatigue; HCV, Hepatitis C virus; HL, Hodgkin lymphoma; ICD, International classification of disease; IQR, interquartile range; LCH, Langerhans cell histiocytosis; LMB, Burkitt lymphoma; NA, data not available; NHL, non‐Hodgkin lymphoma; PCR, polymerase chain reaction; RMS, rhabdomyosarcoma; SD, standard deviation; STS, soft tissue sarcoma.

^a^ Refers to the child's cancer diagnosis.

^b^ Childhood Cancer Survivor Study (CCSS).

^c^ Maintenance and Use of Data for the Study of Hospital Clientele data set, Quebec, Canada.

^d^ Swiss Childhood Cancer Survivor Study (SCCSS).

^e^ Manchester Children's Tumor Registry.

^f^ Dutch Childhood Cancer Survivor Study‐Late Effects After Childhood Cancer (DCCSS‐LATER).

^g^ National Cancer Institute and Five‐Center Study (the Connecticut Tumor Registry, a group of hospitals in California, the Universities of Iowa and Kansas, and MD Anderson Cancer Center, Houston).

^h^ CCSS‐specific cancers include leukemia, CNS tumors, HL, NHL, renal tumors, neuroblastomas, STS, bone tumors.

^i^ Norwegian Family Based Life Course Study (the Cancer Registry, the Medical Birth Registry, Statistics Norway).

^j^ Pediatric Oncology Group of Ontario^'^s Networked Information System (POGONIS).

^k^ Tertiary care centers designated by government policy to treat and hospitalize children with cancer in the province of Québec, Canada.

^l^ Nordic Society of Pediatric Hematology and Oncology (NOPHO)‐AML‐84, ‐88, and ‐93 trials.

^m^ National central population registries—cancer registration.

^n^ Linked national registers in Denmark and Sweden.

Fourteen (32%) studies included a control group: four studies [9, 10, 13, 41] included data from siblings of children without cancer; nine studies [3, 11, 14, 17, 23, 28, 42, 43, 49] included normative or population data; and one study included both [15]. Eighteen (41%) publications included overlapping sibling cohorts from the CCSS [18, 20, 24, 25, 26, 27, 28, 30, 31, 32, 33, 35, 40, 42, 43, 44, 45, 50]. Four (9%) publications were based on the Swiss Childhood Cancer Survivor Study (SCCSS) [14, 15, 37, 38] and four (9%) were based on the Dutch Childhood Cancer Survivor Study—Late effects after childhood cancer (DCCS‐LATER) [6, 17, 21, 36, 39]. The representative publication chosen for each outcome is identified in Table S3. Overall, 13 (30%) studies [3, 9, 10, 13, 16, 23, 28, 29, 41, 42, 43, 47] were focused on siblings or family members of children with cancer as the study population, whereas 29 (66%) studies [11, 12, 17, 18, 19, 20, 21, 22, 24, 25, 26, 27, 30, 31, 32, 33, 34, 35, 36, 37, 38, 39, 40, 44, 45, 46, 48, 49, 51] focused on children with cancer as the study population and siblings were treated as their control group.

The risk of bias assessment resulted in 38 (86%) studies [3, 10, 11, 13, 14, 15, 16, 17, 18, 20, 21, 22, 23, 24, 25, 26, 27, 28, 30, 31, 32, 33, 35, 36, 37, 38, 39, 40, 41, 42, 43, 44, 46, 47, 48, 49, 51, 52] rated as low risk, five (11%) studies [12, 19, 29, 34, 45] as moderate risk, and one (2%) study [9] at high risk (Table S4).

### 3.1 Mortality

Two studies [23, 41] focused on mortality among siblings of children with cancer. One study [23] reported no significant difference in all‐cause mortality incidence between siblings and controls (hazard ratio (HR), 0.73; 95% CI, 0.44–1.3) [23]. The second study [41] reported a greater risk of cancer‐related mortality compared to siblings of healthy children (mortality rate ratio, 2.83; 95% CI, 1.41–5.67) [41].

### 3.2 Cancer

**FIGURE 1:**
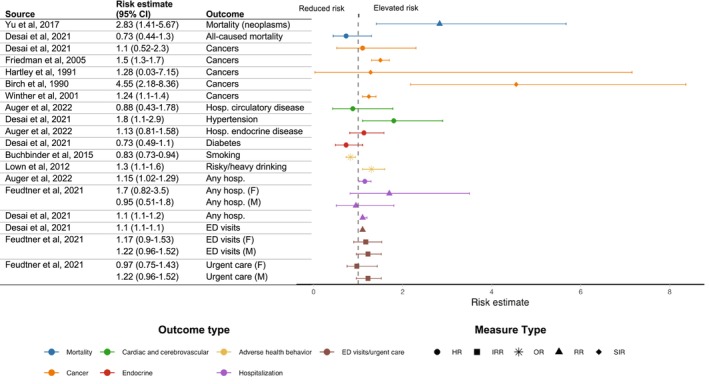
Risk estimates in siblings of children with cancer compared to controls, stratified by outcome types. F, female; HR, hazard ratio; IRR, incidence rate ratio; M, male; OR, odds ratio; RR, rate ratio; SIR, standardized incidence ratio.

Of the 13 studies focused on cancer development among siblings of children with cancer, seven [11, 12, 19, 27, 30, 39, 51] reported measures of frequency and six [16, 22, 23, 28, 29, 47] reported measures of risk. Prevalence of cancer in siblings ranged from 0% (0/56) [12] to 5% (3/60) [19]. Compared to controls, two studies found no statistically significant risk of cancer (HR, 1.1; 95% CI, 0.52–2.3 [23] and standardized incidence ratio (SIR), 1.28; 95% CI, 0.03–7.15 [29]). In contrast, three studies reported an increased cancer risk in siblings compared to controls (SIR, 1.5; 95% CI, 1.3–1.7 [28], SIR, 4.55; 95% CI, 2.18–8.36 [16], and SIR, 1.24; 95% CI, 1.1–1.4 [47] respectively) (Figure 1). A fourth study [22] reported an increased risk of both retinoblastoma (HR, 6.11; 95% CI, 3.09–12.05) and hepatoblastoma (HR, 5.85; 95% CI, 1.70–20.18) in siblings of children with solid tumors compared to controls. After excluding for probable hereditary cancer syndromes, the risk of hepatoblastoma remained significantly elevated (HR, 1.73; 95% CI, 1.07–2.78), whereas the risk of retinoblastoma was still increased, yet not statistically significant (HR, 2.33; 95% CI, 0.88–6.46) [22].

### 3.3 Chronic Organ System Diseases

#### 3.3.1 Neurological

Eight studies [13, 26, 27, 30, 37, 38, 39, 51] evaluated neurological outcomes in siblings. Prevalence of neurological impairment was reported in seven studies [26, 27, 30, 37, 38, 39, 51] ranging from 0.3% (2/534) [37] to 24.5% (78/319) [51]. There was an increased risk of hospitalization for nervous system conditions in siblings compared to controls (HR, 1.57; 95% CI, 1.05–2.35) [13]. Detailed neurological outcomes are reported in Table S3.

#### 3.3.2 Cardiac and Cerebrovascular

Overall, 16 studies [12, 13, 18, 20, 23, 25, 26, 27, 30, 34, 37, 38, 39, 45, 48, 51] reported data on cardiac and cerebrovascular outcomes. Fourteen studies [6, 12, 18, 20, 25, 26, 27, 30, 34, 37, 38, 45, 48, 51] reported measures of frequency of cardiac outcomes.

#### 3.3.3 Hypertension

The prevalence of hypertension in siblings of children with cancer ranged from 3% (raw data not available) [38] to 19.1% (61/319) [51]. One study showed that, compared to controls, siblings were at increased risk of hypertension (HR, 1.8; 95% CI, 1.1–2.9) [23].

#### 3.3.4 Stroke

The prevalence of stroke in siblings of children with cancer ranged from 0% (0/999) [38] to 0.5% (27/5051) [25]. One study reported cumulative incidence for stroke (cumulative incidence, 1.1%; 95% CI, 0.4%–1.7%) [45].

#### 3.3.5 Cardiac Disease

The prevalence of congestive heart failure (CHF) ranged from 0% (0/999) [38] to 0.3% (1/319) [20, 25, 51]. Two studies reported measures of cumulative incidence for ischemic heart disease (cumulative incidence, 1.2%; 95% CI, 0.4–1.7) [45] and congestive heart failure (cumulative incidence, 0.3%; 95% CI, 0.1%–0.5%) [20], respectively. One study [13] assessed the hospitalization rate for circulatory disease, reporting no excess risk (HR, 0.88; 95% CI, 0.43–1.78).

#### 3.3.6 Endocrine

Fourteen studies [11, 12, 13, 23, 25, 26, 27, 30, 33, 38, 39, 44, 48, 51] reported data on endocrine outcomes in siblings of children with cancer. The prevalence of diabetes ranged from 1% (raw data not available) [38] to 3.1% (10/319 and 21/792) [11, 51]. No significant increased risk of diabetes was reported for siblings of children with cancer compared to matched controls (HR, 0.73; 95% CI, 0.49–1.1) in one study [23]. No significant risk of hospitalization for endocrine disease in siblings were reported in one study (HR, 1.13; 95% CI, 0.81–1.58) (Figure 1) [13]. Detailed endocrine outcomes are reported in Table S2.

#### 3.3.7 Pulmonary

Eight studies [11, 12, 13, 30, 34, 38, 39, 50] evaluated pulmonary outcomes in siblings of children with cancer. The prevalence of asthma was 22.1% (153/792) in siblings compared to 18.5% (127/707) in matched controls [11]. There was an increased risk of hospitalization for respiratory disease (HR, 1.18; 95% CI, 1.01–1.40) [13] associated with having a sibling with cancer compared to controls.

### 3.4 Overweight/Obesity

Overall, six studies [15, 17, 31, 33, 35, 38] evaluated overweight status in siblings and all reported prevalence which ranged from 20% (149/725) [15] to 31.1% (913/2936) [33]. Two studies [15, 17] reported data from controls (20% (149/725) in siblings vs. 24% (2285/9591) in controls [15], and 27.4% (raw data not available) in siblings vs. 28.4% (raw data not available) in controls [17]). Ten studies [15, 17, 25, 26, 27, 30, 31, 33, 35, 44] reported the prevalence of obesity among siblings of children with cancer, ranging from 4% (34/725) [15] to 28.5% (593/2097) [30]. Two studies reported data from matched controls (4% (34/725) vs. 6% (603/9591) for controls [15], and 9.4% (raw data not available) vs. 9.3% (raw data not available) for controls [17], relative measures not provided). Among the studies which reported frequency measures of overweight/obesity in siblings of children with cancer and controls, no study included a measure of risk.

### 3.5 Healthcare Service Utilization

#### 3.5.1 Hospitalizations and Emergency Department/Urgent Care Visits

Five studies [3, 13, 23, 34, 49] evaluated data on hospitalization in siblings of children diagnosed with cancer. In one study, the prevalence of any hospitalization among siblings was 40% (34/86) [34]. Another study reported a higher rate of hospitalization in siblings (rate per 100 person‐years, 2.69; 95% CI, 1.01–24.3) compared to controls (rate per 100 person‐years, 1.87; 95% CI, 1.13–3.09) [49]. Two studies [13, 23] found an increased risk of hospitalization associated with having a sibling with cancer (respectively RR, 1.15; 95% CI, 1.02–1.29 [13] and IRR, 1.1; 95% CI, 1.1–1.2 [23]) (Figure 1). The risk of hospitalization was significantly greater for nervous system (HR, 1.57; 95% CI, 1.05–2.35), respiratory (HR, 1.18; 95% CI, 1.01–1.40), digestive (HR, 1.38; 95% CI, 1.09–1.74), and skin (HR, 1.60; 95% CI, 1.13–2.28) disorders in siblings [13]. Children who had a sibling with cancer were at risk of hospitalization for conditions such as pneumonia, inflammatory bowel disease, and other morbidities [13]. One study [3] assessed sisters and brothers independently and found a trend toward increased risk of hospitalization (respectively IRR, 1.7; 95% CI, 0.82–3.5, and 0.95; 95% CI, 0.51–1.8) [3]. Sibling cases were also more likely than controls to receive preventive healthcare, such as undergoing periodic health checkups (OR, 1.1; 95% CI, 1.0–1.1) and receiving influenza vaccinations (OR, 1.5; 95% CI, 1.4–1.6) [23].

Two studies [3, 23] examined rates of emergency department (ED) and urgent care visits in siblings of children with cancer. One study reported that, compared to controls, siblings had higher rates of both low and high acuity ED visits (RR, 1.1; 95% CI, 1.1–1.2) [23]. Another study did not find a higher incidence in sisters and brothers separately (respectively IRR, 1.17; 95% CI, 0.9–1.53, and 1.22; 95% CI, 0.96–1.52) [3], compared to controls (Figure 1).

#### 3.5.2 Prescriptions

Two studies [3, 34] reported prescription use in siblings of children with cancer. One study [34] showed a prevalence of medications of 9% (8/86) in siblings. Another study evaluated incidence rates of all prescriptions (expect mental health prescriptions) and an excessive rate of prescriptions was found in siblings, sisters, and brothers separately (respectively IRR, 1.97; 95% CI, 1.63–2.39, and 1.95; 95% CI, 1.61–2.35) compared to controls [3] (Figure 1).

Additional results are outlined in the online supplement (Appendix S1).

## 4 Discussion

We conducted the first comprehensive systematic review examining adverse physical health outcomes and healthcare service utilization in siblings of children diagnosed with pediatric cancer to elucidate the health vulnerabilities of this population. Our systematic review highlighted potential associations between sibling status and an increased prevalence of cancer, organ system impairment, and high healthcare service utilization. Specifically, in studies comparing siblings to controls, we found trends suggesting increased risks of cancer [28, 29], cancer‐related mortality [41], hypertension [23], and excessive alcohol consumption [43]. Although some studies reported that siblings were at higher risk of hospitalizations [13, 23], ED visits [23], and prescription medication use [3], siblings were also more likely to seek preventive healthcare [23] compared to controls.

Prior work focused on siblings of children with chronic [4] and/or life‐threatening [3] conditions reported increased risks of various adverse health outcomes (traumatic brain injury, overweightness, mortality, healthcare encounters, and medications) [3, 4], supporting our hypothesis that sibling status may confer an increased risk to compromised physical health. Our findings align with prior research that documented an elevated risk of adverse health behaviors and cardiovascular disease [1], as well as a higher likelihood of healthcare prevention behavior [53] in siblings of children with cancer.

Our review identified major gaps in sibling research while providing a framework upon which future sibling‐directed work will be based. Few studies treated the siblings as the population of interest, as the majority of published studies were focused on cancer patients themselves. For instance, most CCSS reports included data on siblings as a control group. As such, these studies centered around outcomes which were selected as relevant to pediatric cancer survivors and were generally biased to highlight siblings as the healthier group compared to cancer survivors. As mentioned in Long et al. [1], studies with a priori sibling aims and selected outcomes relevant to siblings were more likely to report statistically and clinically significant findings for siblings. Moreover, CCSS studies which reported siblings' outcomes tended to compare siblings to nonmatched control groups (the control groups were matched to the childhood cancer survivors and not the siblings), limiting robust risk estimates for siblings. Similarly, most studies did not account for the heterogeneity of the sibling sample in their results (results were not adjusted for sibling characteristics), which further diminished their internal validity [1]. Sibling groups tended to have comparatively lower participation rates (particularly in CCSS studies), hindering the external validity of results and study power.

Given that the majority of studies were centered on the children with cancer and not the siblings, often the available sibling data was simplified or failed to include relevant confounding variables. The studies often did not account for socioeconomic status, attained sibling age, cancer‐related factors, parental health, or genetic susceptibility [54]. Siblings within families with high socioeconomic means have been found to be more likely to use healthcare services, whereas advanced age has been described as a predictor of adverse health behaviors [54]. Determining whether cancer‐related factors (e.g., index child's cancer diagnosis, treatment course, prognosis) significantly impact the sibling's health requires the availability of granular sibling data [55, 56]. For example, a life‐threatening pediatric cancer diagnosis has been associated with greater use of healthcare services in siblings [3]. Conversely, siblings of children with skin cancer were more likely to practice skin cancer prevention [53], raising the question of potential protective factors among siblings.

Family‐related exposures may also influence the physical health of siblings. A cancer diagnosis in a family member deeply disrupts family roles and may limit parental support for the siblings [57]. Siblings can be particularly vulnerable during cancer treatment as a result of parental absence, loss of family routine, frequent transitions between home and hospital, and overall lack of stability [57, 58]. The impact of parental health on sibling health has been poorly studied but likely influences sibling health and/or healthcare service utilization. For example, parental somatization has been found to be a predictor of siblings' symptoms and could result in reporting of sibling health status to a specialist [54]. Shared environmental exposures and genetic susceptibilities are known to be associated with the development of chronic health conditions within family settings and may represent important confounding factors that were largely absent from the published literature on siblings of children with cancer. Modifiable factors such as smoking (including second‐hand cigarette smoke), alcohol consumption, exercise, and nutrition are known contributors to chronic conditions of the cardiovascular system, as well as cancer and metabolic disorders [59, 60]. Other contributors to chronic diseases include radiation exposure, residential location (e.g., near a nuclear facility), and prolonged contact with organic pollutants (e.g., pesticides, industrial, agricultural chemicals), volatile organic compounds (e.g., solvent, fuels), and plastics [59, 60]. For instance, pesticide exposures have been linked to the development of several cancers, including brain, prostate, kidney, non‐Hodgkin lymphoma, and leukemia [61]. Periods of exposure are critical, with elevated risks associated with exposure during prenatal and postnatal periods, as well as with parental occupational exposures [61]. Additionally, only a few included studies [16, 19, 22, 23, 28, 29, 47] considered genetic susceptibility in their findings. Overall, both environmental and genetic factors could account for the development of cancer and other chronic diseases in families [47]. Deciphering the impact of these potential confounding factors on health outcomes in siblings is essential and will require further investigation. Our systematic review therefore highlighted the persistent knowledge gaps surrounding research on siblings of children with cancer, providing direction and opportunities for future work.

This review has several limitations. Although we reported a trend toward increased risk of multiple outcomes among siblings of children with cancer compared to controls, important study heterogeneity rendered study comparisons difficult and meta‐analyses inappropriate. Firstly, we found significant heterogeneity in the populations of interest (relating to both the child with cancer and their siblings). Most studies did not account for the specific cancer diagnosis, stage of the disease, treatment of the index child, and time since diagnosis of the index child. For the siblings, there was heterogeneity regarding their age distribution, length of follow‐up, and timing of outcome assessment. Among the included studies, we also identified varying study designs including population‐based, cross‐sectional, and case–control studies. Each study design may infer unique methodological biases, further supporting our decision to omit meta‐analyses.

Importantly, there was noteworthy heterogeneity in the study outcome measurements and definitions. Data sources included medical records, administrative databases, national health registries, and self‐reported outcomes collected from interviews and surveys. In our systematic review, 72% of the studies included self‐reported data in siblings of children with cancer. Self‐reported data may introduce information biases such as response, reference, and recall biases [62, 63]. The patient's ability to accurately report the occurrence of health outcomes requires validated questionnaires [62]. Self‐reported data may not reliably estimate health conditions compared to other unbiased data sources such as health administrative data [62]. Future work should leverage alternative methods for establishing health outcomes in our population. Additionally, health outcomes were defined differently across studies, introducing outcome‐reporting bias. The selective reporting of heterogeneous outcomes among siblings of children with cancer was a driving factor behind the omission of meta‐analyses in our systematic review [64].

Another limitation of the published literature is that few studies compared the sibling population to matched controls, which prevented comparative measurements and likely contributed to the conflicting results in the literature. Few studies provided longitudinal measurements of outcome assessment and included short follow‐up periods for siblings; most studies were cross‐sectional and retrospective, restricting the long‐term assessment of the impact of childhood cancer on the siblings. The scarcity of normative data for our population hindered the analysis of association. Given the heterogeneous nature of the available data, attempting to extract normative data for calculating association estimates was found to be ineffective. Therefore, risk could not be determined for studies which did not include a matched control population.

Lastly, stratified analyses to determine the impact of specific factors (e.g., sibling attained age, index child's cancer type) on the health outcomes of siblings could not be conducted, as the majority of studies reported pooled sibling data and accessing the primary data was not possible.

Overall, the important heterogeneity in the published sibling literature contributed to the lack of clearly defined health risks in this population. Although these limitations represented barriers to performing meta‐analyses, our extensive and robust literature review provides a solid foundation for future sibling‐directed work. Future research using matched population controls is needed to establish valid comparisons for determining the risks of organ system impairment and healthcare service utilization among siblings of children with cancer. Prospective, longitudinal sibling studies would allow for the characterization of risk and predictive factors, and inference of causal relationships. Specifically, identifying at‐risk subgroups of siblings of children with cancer is imperative for developing informed, preventative recommendations and surveillance strategies. This study therefore lays the groundwork for further investigation to improve healthcare directives and clinical guidelines for the long‐term care of siblings and their families.

## 5 Conclusion

In this systematic review, we identified that siblings of children with cancer may have an increased risk of developing adverse physical health diagnoses and healthcare service utilization. We encountered important study heterogeneity, specifically surrounding populations of interest, study designs, and outcomes of interest, which prevented meta‐analyses. Despite these limitations, the current review underscores the pressing need for further research to improve our understanding of the impact of a pediatric cancer diagnosis on the siblings' long‐term health. Future studies with standardized methodologies would allow for data pooling and a comprehensive analysis of outcome risk development among siblings of children with cancer. A robust understanding of the physical health risks associated with having a sibling with cancer would inform surveillance guidelines and support the development of targeted interventions to mitigate risks.

## Author Contributions

**Victorine Sirveaux:** conceptualization (equal), data curation (lead), formal analysis (lead), investigation (lead), project administration (equal), supervision (equal), visualization (lead), writing – original draft preparation (lead), writing – review and editing (lead). **Lily Puterman‐Salzman:** conceptualization (equal), data curation (equal), investigation (equal), project administration (equal), supervision (equal), writing – original draft preparation (supporting), writing – review and editing (supporting). **Yue Qian Zhang:** data curation (equal), investigation (equal), writing – review and editing (supporting). **Eleni Sotirakos:** data curation (equal), investigation (supporting), writing – review and editing (supporting). **Philippe Dodin:** investigating (supporting), methodology (lead), writing – review and editing (supporting). Guillaume Dumas: methodology (supporting), writing – review and editing (supporting). **Eyal Cohen:** methodology (supporting), writing – review and editing (supporting). **Nadia Roumeliotis:** methodology (supporting), writing – review and editing (supporting). **Petros Pechlivanoglou:** methodology (supporting), writing – review and editing (supporting). **Hallie Coltin:** conceptualization (lead), funding acquisition (lead), investigation (supporting), methodology (equal), project administration (supporting), supervision (lead), writing – original draft preparation (supporting), writing – review and editing (equal).

## Conflicts of Interest

The authors declare no conflicts of interest.

## Supporting information


Data S1

Data S2

## Data Availability

The data that support the findings of this study are available from the corresponding author upon reasonable request. H.C. had full access to all the data in the study and takes responsibility for the integrity of the data and the accuracy of the data analysis.
