# Supplementary material for: Adverse Physical Health Outcomes and Healthcare Service Utilization in Siblings of Children With Cancer: A Systematic Review
Source: Cancer Med. 2025 Jul 25;14(15):e71035. doi: 10.1002/cam4.71035 (PMC12290661; doi:10.1002/cam4.71035)
Supplement: Supplementary file 1 — Data S1 [file CAM4-14-e71035-s001.docx]

Supplemental Online Content

**eFigure 1.** PRISMA flow-diagram of study selection

**eAppendix 1.** PRISMA checklist

**eAppendix 2.** Additional results

**eTable 1.** Search strategy

**eTable 2.** Completed data extraction table

**eTable 3.** Results reported by outcomes

**eTable 4.** Risk of bias assessment (JBI Checklist for Prevalence Studies)

**References**

**eFigure 1. PRISMA flow-diagram of study selection**

**
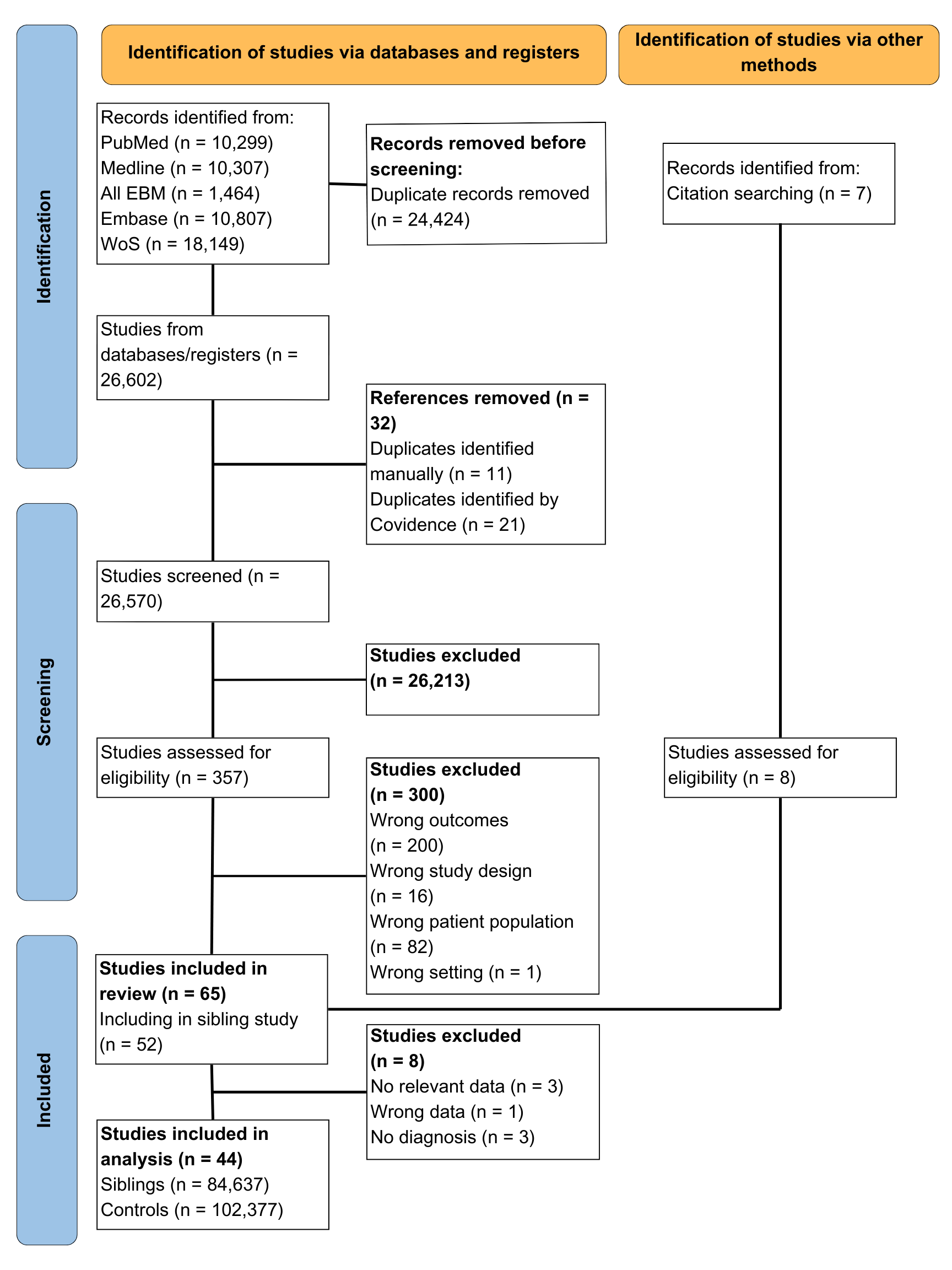
**

**eAppendix 1. PRISMA checklist**

| **Section and Topic** | **Item #** | **Checklist item** | **Location where item is reported** |
| --- | --- | --- | --- |
| **TITLE** | | |  |
| Title | 1 | Identify the report as a systematic review. | 1 |
| **ABSTRACT** | | |  |
| Abstract | 2 | See the PRISMA 2020 for Abstracts checklist. | 4-5 |
| **INTRODUCTION** | | |  |
| Rationale | 3 | Describe the rationale for the review in the context of existing knowledge. | 6 |
| Objectives | 4 | Provide an explicit statement of the objective(s) or question(s) the review addresses. | 6 |
| **METHODS** | | |  |
| Eligibility criteria | 5 | Specify the inclusion and exclusion criteria for the review and how studies were grouped for the syntheses. | 7 |
| Information sources | 6 | Specify all databases, registers, websites, organisations, reference lists and other sources searched or consulted to identify studies. Specify the date when each source was last searched or consulted. | 7 |
| Search strategy | 7 | Present the full search strategies for all databases, registers and websites, including any filters and limits used. | 7, supplemental |
| Selection process | 8 | Specify the methods used to decide whether a study met the inclusion criteria of the review, including how many reviewers screened each record and each report retrieved, whether they worked independently, and if applicable, details of automation tools used in the process. | 8 |
| Data collection process | 9 | Specify the methods used to collect data from reports, including how many reviewers collected data from each report, whether they worked independently, any processes for obtaining or confirming data from study investigators, and if applicable, details of automation tools used in the process. | 8 |
| Data items | 10a | List and define all outcomes for which data were sought. Specify whether all results that were compatible with each outcome domain in each study were sought (e.g. for all measures, time points, analyses), and if not, the methods used to decide which results to collect. | 7, Table 1 |
|  | 10b | List and define all other variables for which data were sought (e.g. participant and intervention characteristics, funding sources). Describe any assumptions made about any missing or unclear information. | Table 2 |
| Study risk of bias assessment | 11 | Specify the methods used to assess risk of bias in the included studies, including details of the tool(s) used, how many reviewers assessed each study and whether they worked independently, and if applicable, details of automation tools used in the process. | 8 |
| Effect measures | 12 | Specify for each outcome the effect measure(s) (e.g. risk ratio, mean difference) used in the synthesis or presentation of results. | 8 |
| Synthesis methods | 13a | Describe the processes used to decide which studies were eligible for each synthesis (e.g. tabulating the study intervention characteristics and comparing against the planned groups for each synthesis (item #5)). | 8 |
|  | 13b | Describe any methods required to prepare the data for presentation or synthesis, such as handling of missing summary statistics, or data conversions. | 8 |
|  | 13c | Describe any methods used to tabulate or visually display results of individual studies and syntheses. | 7 |
|  | 13d | Describe any methods used to synthesize results and provide a rationale for the choice(s). If meta-analysis was performed, describe the model(s), method(s) to identify the presence and extent of statistical heterogeneity, and software package(s) used. | 8 |
|  | 13e | Describe any methods used to explore possible causes of heterogeneity among study results (e.g. subgroup analysis, meta-regression). | NA |
|  | 13f | Describe any sensitivity analyses conducted to assess robustness of the synthesized results. | NA |
| Reporting bias assessment | 14 | Describe any methods used to assess risk of bias due to missing results in a synthesis (arising from reporting biases). | NA |
| Certainty assessment | 15 | Describe any methods used to assess certainty (or confidence) in the body of evidence for an outcome. | NA |
| **RESULTS** | | |  |
| Study selection | 16a | Describe the results of the search and selection process, from the number of records identified in the search to the number of studies included in the review, ideally using a flow diagram. | 8-9 |
|  | 16b | Cite studies that might appear to meet the inclusion criteria, but which were excluded, and explain why they were excluded. | NA |
| Study characteristics | 17 | Cite each included study and present its characteristics. | 9, Table 2 |
| Risk of bias in studies | 18 | Present assessments of risk of bias for each included study. | 9, supplemental |
| Results of individual studies | 19 | For all outcomes, present, for each study: (a) summary statistics for each group (where appropriate) and (b) an effect estimate and its precision (e.g. confidence/credible interval), ideally using structured tables or plots. | 8-14, Figure 1, supplemental |
| Results of syntheses | 20a | For each synthesis, briefly summarise the characteristics and risk of bias among contributing studies. | NA |
|  | 20b | Present results of all statistical syntheses conducted. If meta-analysis was done, present for each the summary estimate and its precision (e.g. confidence/credible interval) and measures of statistical heterogeneity. If comparing groups, describe the direction of the effect. | 8-14 |
|  | 20c | Present results of all investigations of possible causes of heterogeneity among study results. | NA |
|  | 20d | Present results of all sensitivity analyses conducted to assess the robustness of the synthesized results. | NA |
| Reporting biases | 21 | Present assessments of risk of bias due to missing results (arising from reporting biases) for each synthesis assessed. | NA |
| Certainty of evidence | 22 | Present assessments of certainty (or confidence) in the body of evidence for each outcome assessed. | NA |
| **DISCUSSION** | | |  |
| Discussion | 23a | Provide a general interpretation of the results in the context of other evidence. | 14-18 |
|  | 23b | Discuss any limitations of the evidence included in the review. | 18-19 |
|  | 23c | Discuss any limitations of the review processes used. | 20 |
|  | 23d | Discuss implications of the results for practice, policy, and future research. | 20-21 |
| **OTHER INFORMATION** | | |  |
| Registration and protocol | 24a | Provide registration information for the review, including register name and registration number, or state that the review was not registered. | 7 |
|  | 24b | Indicate where the review protocol can be accessed, or state that a protocol was not prepared. | 7 |
|  | 24c | Describe and explain any amendments to information provided at registration or in the protocol. | NA |
| Support | 25 | Describe sources of financial or non-financial support for the review, and the role of the funders or sponsors in the review. | NA |
| Competing interests | 26 | Declare any competing interests of review authors. | NA |
| Availability of data, code and other materials | 27 | Report which of the following are publicly available and where they can be found: template data collection forms; data extracted from included studies; data used for all analyses; analytic code; any other materials used in the review. | NA |

*From:*  Page MJ, McKenzie JE, Bossuyt PM, Boutron I, Hoffmann TC, Mulrow CD, et al. The PRISMA 2020 statement: an updated guideline for reporting systematic reviews. BMJ 2021;372:n71. doi: 10.1136/bmj.n71

**eAppendix 2. Additional results**

**Chronic Organ System Diseases**

***Auditory***

Two studies^1,2^ reported prevalence of auditory conditions in siblings of children with cancer, ranging from 0.6% (primary data not available)^2^ to 6% (6/1066)^1^. Detailed auditory conditions are reported in **eTable 3**.

***Ophthalmologic***
Seven studies^1-7^ reported ophthalmologic outcomes in siblings of children with cancer, with prevalence of cataracts and visual impairments ranging from 0% (0/534)^7^ to 12% (primary data not available)^1^. There was no significant risk of hospitalization for eye or mastoid conditions (HR, 1.44; 95% CI, 0.89-2.32)^3^ in the single study which compared to matched controls.

***Bone/Musculoskeletal***
Of the 9 studies^1-9^ investigating bone or musculoskeletal outcomes in siblings of children with cancer, a prevalence range of 0% (0/999, osteoporosis)^1^ to 13% (primary data not available, any musculoskeletal chronic health conditions (CHC))^1^ was found. One study^3^ included matched controls, found no significant risk of hospitalization for musculoskeletal conditions (HR, 1.10; 95% CI, 0.61-1.97)^3^ associated with having a sibling diagnosed with cancer.

***Amputation***

One study^10^ evaluated limb amputation in siblings of children with cancer, with a prevalence of 0.1% (1/906); controls were not included.

***Renal* and *Hepatic***
Four studies^1,2,9,11^ reported prevalence of renal conditions in siblings, ranging from 0% (0/56, renal failure)^11^ to 7% (primary data not available, any renal CHC)^1^. No study included matched controls, and there was no measure of association between renal conditions and the diagnosis of siblings with cancer.

One study evaluated the prevalence of hepatic outcomes in siblings of children with cancer, with a result of 0.8% (8/1066, hepatic conditions).^2^ There were no matched controls.

***Genitourinary***

One study evaluated genitourinary outcomes in siblings, reporting no increased risk of hospitalization for genitourinary conditions (HR, 1.07; 95% CI, 0.74-1.56)^3^ in siblings compared to controls.

***Digestive/Gastrointestinal***
Four studies^1-4^ assessed gastrointestinal outcomes in siblings of children diagnosed with cancer. Three^1,2,4^ studies reported the prevalence ranging from 0% (0/999, problems with oesophagus)^1^ to 9.1% (29/319, gastrointestinal impairments)^4^. An increased risk of hospitalization for digestive conditions was reported (HR, 1.38; 95% CI, 1.09-1.74)^3^ in one study, compared to matched controls.

**Pain**Eight^1,5-7,12-15^ publications focused on pain outcomes in siblings of children with cancer. The majority of the studies reported prevalence ranging from 0% (0/534, pain/fatigue syndrome)^7^ to 25% (primary data not available, migraines)^6^. One study^13^ evaluated differences in the number of days per patient-years at risk of headache (differences, -1.8; 95% CI %, -2.6 to -1.1 days) and stomach ache (differences, -1.0; 95% CI %, -1.5 to -0.4 days) compared to matched controls.

**Infertility**
Two studies^5,16^ reported data on the prevalence of infertility in siblings, ranging from 8.1% (82/1029, decreased fertility)^5^ to 12.0% (22/185, consultation with a reproductive specialist). In the general population, prevalence rates were similar for decreased fertility and use of fertility services compared to the siblings population (respectively 8.5% vs 8.1% in siblings and 12.2% vs 12.0% in siblings); whereas prevalence of assisted reproductive treatment (ART) in men was elevated in siblings compared to the general population (9.2% vs 0.5%)^17^. No study assessed measures of association between the diagnosis of a sibling/child with cancer and adverse reproductive health outcomes.

**Congenital Anomalies**

Two studies^18,19^ evaluated congenital anomalies or birth defects in siblings. In one study, there was an increased risk, but not statistically significant, of congenital anomalies compared to controls (adjusted OR, 1.54; 95% CI, 0.99-2.41)^19^, most likely attributed to an excess of cardiac anomalies, and a measure of prevalence of 12.4% (61/1015). Prevalence of 14% (12/84) of birth defects in siblings were reported in the second study^18^.

**Comorbidities and infections**

Four studies^1,2,10,20^ reported prevalence of any comorbidity (diagnoses not specified) in siblings, ranging from 0.8% (7/906, comorbidities >2)^10^ to 48% (primary data not available, any CHC)^1^. There were no reported measure of risk comparing siblings of children with cancer to a control population.
One study^21^ reported a prevalence of 4% (12/274) of Hepatitis C virus (HCV) infection in
siblings of children with cancer; controls were not included.

**Adverse Health Behaviors**

Six studies^9,10,22-25^ evaluated smoking tendencies and excessive alcohol consumption in siblings of children diagnosed with cancer. Five^9,10,22,23,25^ reported prevalences ranging from 8.8% (primary data not available, excessive alcohol consumption)^10^ to 47% (106/224, smoking)^23^. One study^10^ reported data in controls (smoking: 13.4% (primary data not available) in siblings vs 22.4% (primary data not available) in controls, and excessive alcohol consumption: 8.8% (primary data not available) in siblings vs 9.2% (primary data not available) in controls). One study^25^ reported an increased risk of risky drinking in siblings compared to controls (OR, 1.3; 95% CI, 1.1-1.6). Another study^24^ showed that siblings were more likely to be former smokers (OR, 1.21; 95% CI, 1.08-1.35), and less likely to be a current smoker (OR, 0.83; 95% CI, 0.73-0.94), compared to controls.

**eTable 1. Search strategy**

## 1. PubMed 21 juin 2024

| #1 | Santé physique | Hospitalization[mh] OR Emergency Service, Hospital[mh] OR emergency medical services[mesh:noexp] OR emergencies[mesh] OR Family Health[mh] OR health[mh:noexp] OR quality of life[mh:noexp] OR Health Status[mh:noexp] OR emergenc*[tiab] OR "Quality of life"[tiab] OR QoL[tiab] OR hrqol[tiab] OR Wellbeing*[tiab] OR Well being*[tiab] OR Physical[tiab] OR General health[tiab] OR Overall health[tiab] OR Poor health[tiab] OR Health outcome*[tiab] OR Health problem*[tiab] OR Health condition*[tiab] OR Health challenge*[tiab] OR Health concern*[tiab] OR Health issue*[tiab] OR Health status[tiab] OR family health[tiab] OR parental health[tiab] OR hospitalizat*[tiab] OR hospitalisat*[tiab] OR health service use[tiab] OR health services use[tiab] OR healthcare use[tiab] OR health care use[tiab] OR health service utilisation[tiab] OR health services utilisation[tiab] OR healthcare utilisation[tiab] OR health care utilisation[tiab] OR health service utilization[tiab] OR health services utilization[tiab] OR healthcare utilization[tiab] OR health care utilization[tiab] | 2 767 835 |
| --- | --- | --- | --- |
| #2 | Famille | Family[mh] OR caregivers[mh] OR parent*[tiab] OR mother*[tiab] OR father*[tiab] OR sibling*[tiab] OR brother*[tiab] OR sister*[tiab] OR famil*[tiab] OR caregiver*[tiab] OR care-giver*[tiab] OR carer[tiab] OR carers[tiab] OR relatives[tiab] | 2 250 498 |
| #3 | Cancer | Neoplasms[MH] OR Medical Oncology[MH] OR "Oncology Service, Hospital"[MH] OR Oncology Nursing[MH] OR Integrative Oncology[MH] OR Cancer Care Facilities[MH] OR Leukostasis[MH] OR Myelodysplastic-Myeloproliferative diseases[MH] OR Cancer Survivors[mh] OR acanthoma[TIAB] OR acanthomas[TIAB] OR acrochordon[TIAB] OR acrochordons[TIAB] OR acrospiroma[TIAB] OR acrospiromas[TIAB] OR adamantinoma[TIAB] OR adamantinomas[TIAB] OR adenoacanthoma[TIAB] OR adenoacanthomas[TIAB] OR adenoameloblastoma[TIAB] OR adenoameloblastomas[TIAB] OR adenocanthoma[TIAB] OR adenocanthomas[TIAB] OR adenocarcinoma[TIAB] OR adenocarcinomas[TIAB] OR adenofibroma[TIAB] OR adenofibromas[TIAB] OR adenolipoma[TIAB] OR adenolipomas[TIAB] OR adenolymphoma[TIAB] OR adenolymphomas[TIAB] OR adenoma[TIAB] OR adenomas[TIAB] OR adenomatoses[TIAB] OR adenomatosis[TIAB] OR adenomatous[TIAB] OR adenomyoepithelioma[TIAB] OR adenomyoepitheliomas[TIAB] OR adenomyoma[TIAB] OR adenomyomas[TIAB] OR adenosarcoma[TIAB] OR adenosarcomas[TIAB] OR adenoses[TIAB] OR adenosis[TIAB] OR aesthesioneuroblastoma[TIAB] OR aesthesioneuroblastomas[TIAB] OR ameloblastoma[TIAB] OR ameloblastomas[TIAB] OR amyloidoses[TIAB] OR amyloidosis[TIAB] OR anaplasia[TIAB] OR anaplasias[TIAB] OR androblastoma[TIAB] OR androblastomas[TIAB] OR angioblastoma[TIAB] OR angioblastomas[TIAB] OR angioendothelioma[TIAB] OR angioendotheliomas[TIAB] OR angioendotheliomatoses[TIAB] OR angioendotheliomatosis[TIAB] OR angiofibroma[TIAB] OR angiofibromas[TIAB] OR angiofibrosarcoma[TIAB] OR angiofibrosarcomas[TIAB] OR angiokeratoma[TIAB] OR angiokeratomas[TIAB] OR angioleiomyoma[TIAB] OR angioleiomyomas[TIAB] OR angiolipoma[TIAB] OR angiolipomas[TIAB] OR angioma[TIAB] OR angiomas[TIAB] OR angiomatoses[TIAB] OR angiomatosis[TIAB] OR angiomyolipoma[TIAB] OR angiomyolipomas[TIAB] OR angiomyoma[TIAB] OR angiomyomas[TIAB] OR angiomyxoma[TIAB] OR angiomyxomas[TIAB] OR angioreticuloma[TIAB] OR angioreticulomas[TIAB] OR angiosarcoma[TIAB] OR angiosarcomas[TIAB] OR apudoma[TIAB] OR apudomas[TIAB] OR argentaffinoma[TIAB] OR argentaffinomas[TIAB] OR arrhenoblastoma[TIAB] OR arrhenoblastomas[TIAB] OR astroblastoma[TIAB] OR astroblastomas[TIAB] OR astrocytoma[TIAB] OR astrocytomas[TIAB] OR astroglioma[TIAB] OR astrogliomas[TIAB] OR atypia[TIAB] OR atypias[TIAB] OR baltoma[TIAB] OR baltomas[TIAB] OR basiloma[TIAB] OR basilomas[TIAB] OR Birt-Hogg-Dube[TIAB] OR blastoma[TIAB] OR blastomas[TIAB] OR branchioma[TIAB] OR branchiomas[TIAB] OR Buschke-Lowenstein[TIAB] OR cachexia[TIAB] OR cachexias[TIAB] OR cancer[TIAB] OR cancerous[TIAB] OR cancers[TIAB] OR carcinogen[TIAB] OR carcinogenesis[TIAB] OR carcinogenic[TIAB] OR carcinogens[TIAB] OR carcinoid[TIAB] OR carcinoma[TIAB] OR carcinomas[TIAB] OR carcinomatoses[TIAB] OR carcinomatosis[TIAB] OR carcinosarcoma[TIAB] OR carcinosarcomas[TIAB] OR cavernoma[TIAB] OR cavernomas[TIAB] OR cementoma[TIAB] OR cementomas[TIAB] OR cerbB2[TIAB] OR ceruminoma[TIAB] OR ceruminomas[TIAB] OR chemodectoma[TIAB] OR chemodectomas[TIAB] OR cherubism[TIAB] OR chloroma[TIAB] OR chloromas[TIAB] OR cholangiocarcinoma[TIAB] OR cholangiocarcinomas[TIAB] OR cholangiohepatoma[TIAB] OR cholangiohepatomas[TIAB] OR cholangioma[TIAB] OR cholangiomas[TIAB] OR cholangiosarcoma[TIAB] OR cholangiosarcomas[TIAB] OR cholesteatoma[TIAB] OR cholesteatomas[TIAB] OR chondroblastoma[TIAB] OR chondroblastomas[TIAB] OR chondroma[TIAB] OR chondromas[TIAB] OR chondrosarcoma[TIAB] OR chondrosarcomas[TIAB] OR chordoma[TIAB] OR chordomas[TIAB] OR chorioadenoma[TIAB] OR chorioadenomas[TIAB] OR chorioangioma[TIAB] OR chorioangiomas[TIAB] OR choriocarcinoma[TIAB] OR choriocarcinomas[TIAB] OR chorioepithelioma[TIAB] OR chorioepitheliomas[TIAB] OR chorionepithelioma[TIAB] OR chorionepitheliomas[TIAB] OR choristoma[TIAB] OR choristomas[TIAB] OR chromaffinoma[TIAB] OR chromaffinomas[TIAB] OR cocarcinogeneses[TIAB] OR cocarcinogenesis[TIAB] OR collagenoma[TIAB] OR collagenomas[TIAB] OR comedocarcinoma[TIAB] OR comedocarcinomas[TIAB] OR condyloma[TIAB] OR condylomas[TIAB] OR corticotropinoma[TIAB] OR corticotropinomas[TIAB] OR craniopharyngioma[TIAB] OR craniopharyngiomas[TIAB] OR cylindroma[TIAB] OR cylindromas[TIAB] OR cyst[TIAB] OR cystadenocarcinoma[TIAB] OR cystadenocarcinomas[TIAB] OR cystadenofibroma[TIAB] OR cystadenofibromas[TIAB] OR cystadenoma[TIAB] OR cystadenomas[TIAB] OR cystoma[TIAB] OR cystomas[TIAB] OR cystosarcoma[TIAB] OR cystosarcomas[TIAB] OR cysts[TIAB] OR dentinoma[TIAB] OR dentinomas[TIAB] OR dermatofibroma[TIAB] OR dermatofibromas[TIAB] OR dermatofibrosarcoma[TIAB] OR dermatofibrosarcomas[TIAB] OR dermoid[TIAB] OR desmoid[TIAB] OR desmoplastic[TIAB] OR dictyoma[TIAB] OR dictyomas[TIAB] OR dysgerminoma[TIAB] OR dysgerminomas[TIAB] OR dyskeratoma[TIAB] OR dyskeratomas[TIAB] OR dysmyelopoieses[TIAB] OR dysmyelopoiesis[TIAB] OR dysplasia[TIAB] OR dysplastic[TIAB] OR ectomesenchymoma[TIAB] OR ectomesenchymomas[TIAB] OR elastofibroma[TIAB] OR elastofibromas[TIAB] OR enchondroma[TIAB] OR enchondromas[TIAB] OR enchondromatoses[TIAB] OR enchondromatosis[TIAB] OR endothelioma[TIAB] OR endotheliomas[TIAB] OR ependymoblastoma[TIAB] OR ependymoblastomas[TIAB] OR ependymoma[TIAB] OR ependymomas[TIAB] OR epidermoid[TIAB] OR epithelioma[TIAB] OR epitheliomas[TIAB] OR erythroleukaemia[TIAB] OR erythroleukaemias[TIAB] OR erythroleukemia[TIAB] OR erythroleukemias[TIAB] OR erythroplakia[TIAB] OR erythroplakias[TIAB] OR erythroplasia[TIAB] OR erythroplasias[TIAB] OR esthesioneuroblastoma[TIAB] OR esthesioneuroblastomas[TIAB] OR esthesioneuroepithelioma[TIAB] OR esthesioneuroepitheliomas[TIAB] OR exostoses[TIAB] OR exostosis[TIAB] OR fibroadenoma[TIAB] OR fibroadenomas[TIAB] OR fibroadenosarcoma[TIAB] OR fibroadenosarcomas[TIAB] OR fibroadenoses[TIAB] OR fibroadenosis[TIAB] OR fibrochondrosarcoma[TIAB] OR fibrochondrosarcomas[TIAB] OR fibroelastoma[TIAB] OR fibroelastomas[TIAB] OR fibroepithelioma[TIAB] OR fibroepitheliomas[TIAB] OR fibrofolliculoma[TIAB] OR fibrofolliculomas[TIAB] OR fibroid[TIAB] OR fibroids[TIAB] OR fibrolipoma[TIAB] OR fibrolipomas[TIAB] OR fibroliposarcoma[TIAB] OR fibroliposarcomas[TIAB] OR fibroma[TIAB] OR fibromas[TIAB] OR fibromatoses[TIAB] OR fibromatosis[TIAB] OR fibromyoma[TIAB] OR fibromyomas[TIAB] OR fibromyxolipoma[TIAB] OR fibromyxolipomas[TIAB] OR fibromyxoma[TIAB] OR fibromyxomas[TIAB] OR fibroodontoma[TIAB] OR fibroodontomas[TIAB] OR fibrosarcoma[TIAB] OR fibrosarcomas[TIAB] OR fibrothecoma[TIAB] OR fibrothecomas[TIAB] OR fibroxanthoma[TIAB] OR fibroxanthomas[TIAB] OR fibroxanthosarcoma[TIAB] OR fibroxanthosarcomas[TIAB] OR ganglioblastoma[TIAB] OR ganglioblastomas[TIAB] OR gangliocytoma[TIAB] OR gangliocytomas[TIAB] OR ganglioglioma[TIAB] OR gangliogliomas[TIAB] OR ganglioneuroblastoma[TIAB] OR ganglioneuroblastomas[TIAB] OR ganglioneurofibroma[TIAB] OR ganglioneurofibromas[TIAB] OR ganglioneuroma[TIAB] OR ganglioneuromas[TIAB] OR gastrinoma[TIAB] OR gastrinomas[TIAB] OR germinoma[TIAB] OR germinomas[TIAB] OR glioblastoma[TIAB] OR glioblastomas[TIAB] OR gliofibroma[TIAB] OR gliofibromas[TIAB] OR glioma[TIAB] OR gliomas[TIAB] OR gliomatoses[TIAB] OR gliomatosis[TIAB] OR glioneuroma[TIAB] OR glioneuromas[TIAB] OR gliosarcoma[TIAB] OR gliosarcomas[TIAB] OR glomangioma[TIAB] OR glomangiomas[TIAB] OR glomangiomatoses[TIAB] OR glomangiomatosis[TIAB] OR glomangiomyoma[TIAB] OR glomangiomyomas[TIAB] OR glomangiosarcoma[TIAB] OR glomangiosarcomas[TIAB] OR glucagonoma[TIAB] OR glucagonomas[TIAB] OR gonadoblastoma[TIAB] OR gonadoblastomas[TIAB] OR gonocytoma[TIAB] OR gonocytomas[TIAB] OR granuloma[TIAB] OR granulomas[TIAB] OR granulomatoses[TIAB] OR granulomatosis[TIAB] OR gynaecomastia[TIAB] OR gynaecomastias[TIAB] OR gynandroblastoma[TIAB] OR gynandroblastomas[TIAB] OR gynecomastia[TIAB] OR gynecomastias[TIAB] OR haemangioblastoma[TIAB] OR haemangioblastomas[TIAB] OR haemangioma[TIAB] OR haemangiomas[TIAB] OR haemangiopericytoma[TIAB] OR haemangiopericytomas[TIAB] OR haemangiosarcoma[TIAB] OR haemangiosarcomas[TIAB] OR hamartoma[TIAB] OR hamartomas[TIAB] OR hemangioblastoma[TIAB] OR hemangioblastomas[TIAB] OR hemangioendothelioma[TIAB] OR hemangioendotheliomas[TIAB] OR hemangioendotheliosarcoma[TIAB] OR hemangioendotheliosarcomas[TIAB] OR hemangioma[TIAB] OR hemangiomas[TIAB] OR hemangiomatoses[TIAB] OR hemangiomatosis[TIAB] OR hemangiopericytoma[TIAB] OR hemangiopericytomas[TIAB] OR hemangioperithelioma[TIAB] OR hemangioperitheliomas[TIAB] OR hemangiosarcoma[TIAB] OR hemangiosarcomas[TIAB] OR hepatoblastoma[TIAB] OR hepatoblastomas[TIAB] OR hepatocarcinoma[TIAB] OR hepatocarcinomas[TIAB] OR hepatocholangiocarcinoma[TIAB] OR hepatocholangiocarcinomas[TIAB] OR hepatoma[TIAB] OR hepatomas[TIAB] OR hibernoma[TIAB] OR hibernomas[TIAB] OR hidradenoma[TIAB] OR hidradenomas[TIAB] OR hidrocystoma[TIAB] OR hidrocystomas[TIAB] OR histiocytoma[TIAB] OR histiocytomas[TIAB] OR hodgkin[TIAB] OR hodgkins[TIAB] OR hydatidiform[TIAB] OR hydradenoma[TIAB] OR hydradenomas[TIAB] OR hypernephroma[TIAB] OR hypernephromas[TIAB] OR immunocytoma[TIAB] OR immunocytomas[TIAB] OR insulinoma[TIAB] OR insulinomas[TIAB] OR kasabach-merritt[TIAB] OR keratoacanthoma[TIAB] OR keratoacanthomas[TIAB] OR keratoses[TIAB] OR keratosis[TIAB] OR leiomyoblastoma[TIAB] OR leiomyoblastomas[TIAB] OR leiomyofibroma[TIAB] OR leiomyofibromas[TIAB] OR leiomyoma[TIAB] OR leiomyomas[TIAB] OR leiomyomatoses[TIAB] OR leiomyomatosis[TIAB] OR leiomyosarcoma[TIAB] OR leiomyosarcomas[TIAB] OR leukaemia[TIAB] OR leukaemias[TIAB] OR leukemia[TIAB] OR leukemias[TIAB] OR leukoplakia[TIAB] OR leukoplakias[TIAB] OR Leukostases[TIAB] OR Leukostasis[TIAB] OR li fraumeni[TIAB] OR lipoadenoma[TIAB] OR lipoadenomas[TIAB] OR lipoblastoma[TIAB] OR lipoblastomas[TIAB] OR lipoblastomatoses[TIAB] OR lipoblastomatosis[TIAB] OR lipoma[TIAB] OR lipomas[TIAB] OR lipomatoses[TIAB] OR lipomatosis[TIAB] OR liposarcoma[TIAB] OR liposarcomas[TIAB] OR luteinoma[TIAB] OR luteinomas[TIAB] OR luteoma[TIAB] OR luteomas[TIAB] OR lymphangioendothelioma[TIAB] OR lymphangioendotheliomas[TIAB] OR lymphangioleiomyomatoses[TIAB] OR lymphangioleiomyomatosis[TIAB] OR lymphangioma[TIAB] OR lymphangiomas[TIAB] OR lymphangiomatoses[TIAB] OR lymphangiomatosis[TIAB] OR lymphangiomyoma[TIAB] OR lymphangiomyomas[TIAB] OR lymphangiomyomatoses[TIAB] OR lymphangiomyomatosis[TIAB] OR lymphangiosarcoma[TIAB] OR lymphangiosarcomas[TIAB] OR lymphoepithelioma[TIAB] OR lymphoepitheliomas[TIAB] OR lymphoma[TIAB] OR lymphomas[TIAB] OR lymphoproliferation[TIAB] OR lymphoproliferations[TIAB] OR lymphoproliferative[TIAB] OR macroglobulinemia[TIAB] OR macroglobulinemias[TIAB] OR macroprolactinoma[TIAB] OR macroprolactinomas[TIAB] OR malignancies[TIAB] OR malignancy[TIAB] OR malignant[TIAB] OR maltoma[TIAB] OR maltomas[TIAB] OR masculinovoblastoma[TIAB] OR masculinovoblastomas[TIAB] OR mastocytoma[TIAB] OR mastocytomas[TIAB] OR mastocytoses[TIAB] OR mastocytosis[TIAB] OR mcf-7[TIAB] OR medulloblastoma[TIAB] OR medulloblastomas[TIAB] OR medullocytoma[TIAB] OR medullocytomas[TIAB] OR medulloepithelioma[TIAB] OR medulloepitheliomas[TIAB] OR medullomyoblastoma[TIAB] OR medullomyoblastomas[TIAB] OR melanoacanthoma[TIAB] OR melanoacanthomas[TIAB] OR melanoameloblastoma[TIAB] OR melanoameloblastomas[TIAB] OR melanocytoma[TIAB] OR melanocytomas[TIAB] OR melanoma[TIAB] OR melanomas[TIAB] OR melanomatoses[TIAB] OR melanomatosis[TIAB] OR meningioblastoma[TIAB] OR meningioblastomas[TIAB] OR meningioma[TIAB] OR meningiomas[TIAB] OR meningiomatoses[TIAB] OR meningiomatosis[TIAB] OR mesenchymoma[TIAB] OR mesenchymomas[TIAB] OR mesonephroma[TIAB] OR mesonephromas[TIAB] OR mesothelioma[TIAB] OR mesotheliomas[TIAB] OR metaplasia[TIAB] OR metastases[TIAB] OR metastasis[TIAB] OR metastatic[TIAB] OR microcarcinoma[TIAB] OR microcarcinomas[TIAB] OR microglioma[TIAB] OR microgliomas[TIAB] OR micrometastases[TIAB] OR micrometastasis[TIAB] OR mucosites[TIAB] OR mucositis[TIAB] OR myelodysplasia[TIAB] OR myelodysplasias[TIAB] OR myelodysplastic[TIAB] OR myelofibroses[TIAB] OR myelofibrosis[TIAB] OR myelolipoma[TIAB] OR myelolipomas[TIAB] OR myeloma[TIAB] OR myelomas[TIAB] OR myelomatoses[TIAB] OR myelomatosis[TIAB] OR myeloproliferation[TIAB] OR myeloproliferations[TIAB] OR myeloproliferative[TIAB] OR mycosis fungoides[TIAB] OR myoblastoma[TIAB] OR myoblastomas[TIAB] OR myoepithelioma[TIAB] OR myoepitheliomas[TIAB] OR myofibroblastoma[TIAB] OR myofibroblastomas[TIAB] OR myofibroma[TIAB] OR myofibromas[TIAB] OR myofibromatoses[TIAB] OR myofibromatosis[TIAB] OR myofibrosarcoma[TIAB] OR myofibrosarcomas[TIAB] OR myolipoma[TIAB] OR myolipomas[TIAB] OR myoma[TIAB] OR myomas[TIAB] OR myopericytoma[TIAB] OR myopericytomas[TIAB] OR myosarcoma[TIAB] OR myosarcomas[TIAB] OR myxofibroma[TIAB] OR myxofibromas[TIAB] OR myxolipoma[TIAB] OR myxolipomas[TIAB] OR myxoliposarcoma[TIAB] OR myxoliposarcomas[TIAB] OR myxoma[TIAB] OR myxomas[TIAB] OR neoplasia[TIAB] OR neoplasias[TIAB] OR neoplasm[TIAB] OR neoplasms[TIAB] OR neoplastic[TIAB] OR nephroblastoma[TIAB] OR nephroblastomas[TIAB] OR neurilemmoma[TIAB] OR neurilemmomas[TIAB] OR neurilemmomatoses[TIAB] OR neurilemmomatosis[TIAB] OR neurilemoma[TIAB] OR neurilemomas[TIAB] OR neurinoma[TIAB] OR neurinomas[TIAB] OR neuroblastoma[TIAB] OR neuroblastomas[TIAB] OR neurocytoma[TIAB] OR neurocytomas[TIAB] OR neuroepithelioma[TIAB] OR neuroepitheliomas[TIAB] OR neurofibroma[TIAB] OR neurofibromas[TIAB] OR neurofibromatoses[TIAB] OR neurofibromatosis[TIAB] OR neurofibrosarcoma[TIAB] OR neurofibrosarcomas[TIAB] OR neurolipocytoma[TIAB] OR neurolipocytomas[TIAB] OR neuroma[TIAB] OR neuromas[TIAB] OR neuronevus[TIAB] OR neurothekeoma[TIAB] OR neurothekeomas[TIAB] OR nevus[TIAB] OR nonhodgkin[TIAB] OR nonhodgkins[TIAB] OR nonseminoma[TIAB] OR nonseminomas[TIAB] OR nonseminomatous[TIAB] OR odontoameloblastoma[TIAB] OR odontoameloblastomas[TIAB] OR odontoma[TIAB] OR odontomas[TIAB] OR oligoastrocytoma[TIAB] OR oligoastrocytomas[TIAB] OR oligodendroglioma[TIAB] OR oligodendrogliomas[TIAB] OR oncocytoma[TIAB] OR oncocytomas[TIAB] OR oncogen[TIAB] OR oncogene[TIAB] OR oncogenes[TIAB] OR oncogeneses[TIAB] OR oncogenesis[TIAB] OR oncogenic[TIAB] OR oncogens[TIAB] OR oncologic[TIAB] OR oncologist[TIAB] OR oncologists[TIAB] OR oncology[TIAB] OR opsoclonus-myoclonus[TIAB] OR orchioblastoma[TIAB] OR orchioblastomas[TIAB] OR osteoblastoma[TIAB] OR osteoblastomas[TIAB] OR osteochondroma[TIAB] OR osteochondromas[TIAB] OR osteochondrosarcoma[TIAB] OR osteochondrosarcomas[TIAB] OR osteoclastoma[TIAB] OR osteoclastomas[TIAB] OR osteofibrosarcoma[TIAB] OR osteofibrosarcomas[TIAB] OR osteoma[TIAB] OR osteomas[TIAB] OR osteosarcoma[TIAB] OR osteosarcomas[TIAB] OR pancreatoblastoma[TIAB] OR pancreatoblastomas[TIAB] OR papilloma[TIAB] OR papillomas[TIAB] OR papillomata[TIAB] OR papillomatas[TIAB] OR papillomatoses[TIAB] OR papillomatosis[TIAB] OR parachordoma[TIAB] OR parachordomas[TIAB] OR paraganglioma[TIAB] OR paragangliomas[TIAB] OR paraneoplastic[TIAB] OR perineurioma[TIAB] OR perineuriomas[TIAB] OR phaeochromocytoma[TIAB] OR phaeochromocytomas[TIAB] OR pheochromoblastoma[TIAB] OR pheochromoblastomas[TIAB] OR pheochromocytoma[TIAB] OR pheochromocytomas[TIAB] OR pilomatricoma[TIAB] OR pilomatricomas[TIAB] OR pilomatrixoma[TIAB] OR pilomatrixomas[TIAB] OR pinealblastoma[TIAB] OR pinealblastomas[TIAB] OR pinealoblastoma[TIAB] OR pinealoblastomas[TIAB] OR pinealoma[TIAB] OR pinealomas[TIAB] OR pineoblastoma[TIAB] OR pineoblastomas[TIAB] OR pineocytoma[TIAB] OR pineocytomas[TIAB] OR plasmacytoma[TIAB] OR plasmacytomas[TIAB] OR pneumoblastoma[TIAB] OR pneumoblastomas[TIAB] OR pneumocytoma[TIAB] OR pneumocytomas[TIAB] OR polyembryoma[TIAB] OR polyembryomas[TIAB] OR polyhistioma[TIAB] OR polyhistiomas[TIAB] OR polyp[TIAB] OR polyposes[TIAB] OR polyposis[TIAB] OR polyps[TIAB] OR porocarcinoma[TIAB] OR porocarcinomas[TIAB] OR poroma[TIAB] OR poromas[TIAB] OR precancer[TIAB] OR precancerous[TIAB] OR precancers[TIAB] OR preleukaemia[TIAB] OR preleukaemias[TIAB] OR preleukemia[TIAB] OR preleukemias[TIAB] OR premalignant[TIAB] OR preneoplastic[TIAB] OR prolactinoma[TIAB] OR prolactinomas[TIAB] OR protooncogene[TIAB] OR protooncogenes[TIAB] OR pseudotumor[TIAB] OR pseudotumors[TIAB] OR pseudotumour[TIAB] OR pseudotumours[TIAB] OR reninoma[TIAB] OR reninomas[TIAB] OR reticuloendothelioma[TIAB] OR reticuloendotheliomas[TIAB] OR reticulohistiocytoma[TIAB] OR reticulohistiocytomas[TIAB] OR reticuloses[TIAB] OR reticulosis[TIAB] OR retinoblastoma[TIAB] OR retinoblastomas[TIAB] OR rhabdomyoma[TIAB] OR rhabdomyomas[TIAB] OR rhabdomyosarcoma[TIAB] OR rhabdomyosarcomas[TIAB] OR rhabdosarcoma[TIAB] OR rhabdosarcomas[TIAB] OR sarcoma[TIAB] OR sarcomas[TIAB] OR sarcomatoses[TIAB] OR sarcomatosis[TIAB] OR schwannoma[TIAB] OR schwannomas[TIAB] OR schwannomatoses[TIAB] OR schwannomatosis[TIAB] OR seminoma[TIAB] OR seminomas[TIAB] OR seminomatous[TIAB] OR sezary syndrome[TIAB] OR somatostatinoma[TIAB] OR somatostatinomas[TIAB] OR somatotropinoma[TIAB] OR somatotropinomas[TIAB] OR spermatocytoma[TIAB] OR spermatocytomas[TIAB] OR spiradenoma[TIAB] OR spiradenomas[TIAB] OR spongioblastoma[TIAB] OR spongioblastomas[TIAB] OR steatocystoma[TIAB] OR steatocystomas[TIAB] OR subependymoma[TIAB] OR subependymomas[TIAB] OR syringadenoma[TIAB] OR syringadenomas[TIAB] OR syringocystadenoma[TIAB] OR syringocystadenomas[TIAB] OR syringoma[TIAB] OR syringomas[TIAB] OR teratocarcinoma[TIAB] OR teratocarcinomas[TIAB] OR teratoma[TIAB] OR teratomas[TIAB] OR thecoma[TIAB] OR thecomas[TIAB] OR thymolipoma[TIAB] OR thymolipomas[TIAB] OR thymoma[TIAB] OR thymomas[TIAB] OR trichilemmoma[TIAB] OR trichilemmomas[TIAB] OR trichoadenoma[TIAB] OR trichoadenomas[TIAB] OR trichoblastoma[TIAB] OR trichoblastomas[TIAB] OR trichodiscoma[TIAB] OR trichodiscomas[TIAB] OR trichoepithelioma[TIAB] OR trichoepitheliomas[TIAB] OR trichofolliculoma[TIAB] OR trichofolliculomas[TIAB] OR tricholemmoma[TIAB] OR tricholemmomas[TIAB] OR tumor[TIAB] OR tumorgeneses[TIAB] OR tumorgenesis[TIAB] OR tumorgenic[TIAB] OR tumorigeneses[TIAB] OR tumorigenesis[TIAB] OR tumorigenic[TIAB] OR tumorogeneses[TIAB] OR tumorogenesis[TIAB] OR tumorogenic[TIAB] OR tumors[TIAB] OR tumour[TIAB] OR tumours[TIAB] OR vipoma[TIAB] OR vipomas[TIAB] OR waldenstrom[TIAB] OR waldenstroms[TIAB] OR xanthoastrocytoma[TIAB] OR xanthoastrocytomas[TIAB] OR xanthofibroma[TIAB] OR xanthofibromas[TIAB] OR xanthogranuloma[TIAB] OR xanthogranulomas[TIAB] OR xanthoma[TIAB] OR xanthomas[TIAB] OR xanthosarcoma[TIAB] OR xanthosarcomas[TIAB] | 5 812 164 |
| #4 | Pédiatrique | Infant[MH] OR Child[MH] OR Adolescent[MH] OR Intensive Care, Neonatal[MH] OR Intensive Care Units, Neonatal[MH] OR Intensive Care Units, Pediatric[MH] OR Hospitals, Pediatric[MH] OR Nurses, Pediatric[MH] OR Pediatrics[MH] OR Pediatricians[MH] OR Child, Hospitalized[MH] OR Adolescent, Hospitalized[MH] OR newborn*[TIAB] OR new born*[TIAB] OR babie*[TIAB] OR baby*[TIAB] OR infant*[TIAB] OR infancy[TIAB] OR toddler*[TIAB] OR preschool*[TIAB] OR pre school*[TIAB] OR child[TIAB] OR child'[TIAB] OR childs[TIAB] OR child's[TIAB] OR children*[TIAB] OR childhood*[TIAB] OR kid[TIAB] OR kid'[TIAB] OR kids[TIAB] OR kid's[TIAB] OR boy[TIAB] OR boy'[TIAB] OR boys[TIAB] OR boy's[TIAB] OR girl[TIAB] OR girl'[TIAB] OR girls[TIAB] OR girl's[TIAB] OR schoolchild*[TIAB] OR juvenil*[TIAB] OR preadolescen*[TIAB] OR youth*[TIAB] OR adolescen*[TIAB] OR teen[TIAB] OR teen'[TIAB] OR teens[TIAB] OR teen's[TIAB] OR teenage*[TIAB] OR puber[TIAB] OR puber'[TIAB] OR pubers[TIAB] OR puber's[TIAB] OR pubert*[TIAB] OR pubescen*[TIAB] OR high school*[TIAB] OR highschool*[TIAB] OR secondary school*[TIAB] OR paediatric*[TIAB] OR pediatric*[TIAB] OR PICU*[TIAB] OR neonat*[TIAB] OR neo nat*[TIAB] | 4 997 648 |
| #5 | NOT | (Animals[mh] NOT humans[mh]) OR (guinea[TIAB] OR Rat[TIAB] OR Rats[TIAB] OR Mice[TIAB] OR Mouse[TIAB] OR Murine[TIAB] OR Animal*[TIAB] OR veterinar*[TIAB]) | 6 584 608 |
| #6 | Combinaison | ((#1 AND #2 AND #3 AND #4) NOT #5) AND (French[la] OR English[la]) | 10 299 |

## 2. Ovid Medline(R) All 21 juin 2024

| 1 | Santé physique | Exp Hospitalization/ OR Exp Emergency Service, Hospital/ OR emergency medical services/ OR exp emergencies/ OR Exp Family Health/ OR health/ OR quality of life/ OR Health Status/ OR (emergenc* OR "Quality of life" OR QoL OR hrqol OR Wellbeing* OR Well being* OR Physical OR General health OR Overall health OR Poor health OR Health outcome* OR Health problem* OR Health condition* OR Health challenge* OR Health concern* OR Health issue* OR Health status OR family health OR parental health OR hospitalizat* OR hospitalisat* OR "health service use" OR "health services use" OR "healthcare use" OR "health care use" OR health service utilisation OR health services utilisation OR healthcare utilisation OR health care utilisation OR health service utilization OR health services utilization OR healthcare utilization OR health care utilization).ti,ab,kw,kf | 2 779 794 |
| --- | --- | --- | --- |
| 2 | Famille | Exp Family/ OR Exp caregivers/ OR (parent* OR mother* OR father* OR sibling* OR brother* OR sister* OR famil* OR caregiver* OR care-giver* OR carer OR carers OR relatives).ti,ab,kw,kf | 2 251 954 |
| 3 | Cancer | Exp Neoplasms/ OR Exp Medical Oncology/ OR Exp Oncology Service, Hospital/ OR Exp Oncology Nursing/ OR Exp Integrative Oncology/ OR Exp Cancer Care Facilities/ OR Exp Leukostasis/ OR Exp Myelodysplastic-Myeloproliferative diseases/ OR Exp Cancer Survivors/ OR (acanthoma OR acanthomas OR acrochordon OR acrochordons OR acrospiroma OR acrospiromas OR adamantinoma OR adamantinomas OR adenoacanthoma OR adenoacanthomas OR adenoameloblastoma OR adenoameloblastomas OR adenocanthoma OR adenocanthomas OR adenocarcinoma OR adenocarcinomas OR adenofibroma OR adenofibromas OR adenolipoma OR adenolipomas OR adenolymphoma OR adenolymphomas OR adenoma OR adenomas OR adenomatoses OR adenomatosis OR adenomatous OR adenomyoepithelioma OR adenomyoepitheliomas OR adenomyoma OR adenomyomas OR adenosarcoma OR adenosarcomas OR adenoses OR adenosis OR aesthesioneuroblastoma OR aesthesioneuroblastomas OR ameloblastoma OR ameloblastomas OR amyloidoses OR amyloidosis OR anaplasia OR anaplasias OR androblastoma OR androblastomas OR angioblastoma OR angioblastomas OR angioendothelioma OR angioendotheliomas OR angioendotheliomatoses OR angioendotheliomatosis OR angiofibroma OR angiofibromas OR angiofibrosarcoma OR angiofibrosarcomas OR angiokeratoma OR angiokeratomas OR angioleiomyoma OR angioleiomyomas OR angiolipoma OR angiolipomas OR angioma OR angiomas OR angiomatoses OR angiomatosis OR angiomyolipoma OR angiomyolipomas OR angiomyoma OR angiomyomas OR angiomyxoma OR angiomyxomas OR angioreticuloma OR angioreticulomas OR angiosarcoma OR angiosarcomas OR apudoma OR apudomas OR argentaffinoma OR argentaffinomas OR arrhenoblastoma OR arrhenoblastomas OR astroblastoma OR astroblastomas OR astrocytoma OR astrocytomas OR astroglioma OR astrogliomas OR atypia OR atypias OR baltoma OR baltomas OR basiloma OR basilomas OR Birt-Hogg-Dube OR blastoma OR blastomas OR branchioma OR branchiomas OR Buschke-Lowenstein OR cachexia OR cachexias OR cancer OR cancerous OR cancers OR carcinogen OR carcinogenesis OR carcinogenic OR carcinogens OR carcinoid OR carcinoma OR carcinomas OR carcinomatoses OR carcinomatosis OR carcinosarcoma OR carcinosarcomas OR cavernoma OR cavernomas OR cementoma OR cementomas OR cerbB2 OR ceruminoma OR ceruminomas OR chemodectoma OR chemodectomas OR cherubism OR chloroma OR chloromas OR cholangiocarcinoma OR cholangiocarcinomas OR cholangiohepatoma OR cholangiohepatomas OR cholangioma OR cholangiomas OR cholangiosarcoma OR cholangiosarcomas OR cholesteatoma OR cholesteatomas OR chondroblastoma OR chondroblastomas OR chondroma OR chondromas OR chondrosarcoma OR chondrosarcomas OR chordoma OR chordomas OR chorioadenoma OR chorioadenomas OR chorioangioma OR chorioangiomas OR choriocarcinoma OR choriocarcinomas OR chorioepithelioma OR chorioepitheliomas OR chorionepithelioma OR chorionepitheliomas OR choristoma OR choristomas OR chromaffinoma OR chromaffinomas OR cocarcinogeneses OR cocarcinogenesis OR collagenoma OR collagenomas OR comedocarcinoma OR comedocarcinomas OR condyloma OR condylomas OR corticotropinoma OR corticotropinomas OR craniopharyngioma OR craniopharyngiomas OR cylindroma OR cylindromas OR cyst OR cystadenocarcinoma OR cystadenocarcinomas OR cystadenofibroma OR cystadenofibromas OR cystadenoma OR cystadenomas OR cystoma OR cystomas OR cystosarcoma OR cystosarcomas OR cysts OR dentinoma OR dentinomas OR dermatofibroma OR dermatofibromas OR dermatofibrosarcoma OR dermatofibrosarcomas OR dermoid OR desmoid OR desmoplastic OR dictyoma OR dictyomas OR dysgerminoma OR dysgerminomas OR dyskeratoma OR dyskeratomas OR dysmyelopoieses OR dysmyelopoiesis OR dysplasia OR dysplastic OR ectomesenchymoma OR ectomesenchymomas OR elastofibroma OR elastofibromas OR enchondroma OR enchondromas OR enchondromatoses OR enchondromatosis OR endothelioma OR endotheliomas OR ependymoblastoma OR ependymoblastomas OR ependymoma OR ependymomas OR epidermoid OR epithelioma OR epitheliomas OR erythroleukaemia OR erythroleukaemias OR erythroleukemia OR erythroleukemias OR erythroplakia OR erythroplakias OR erythroplasia OR erythroplasias OR esthesioneuroblastoma OR esthesioneuroblastomas OR esthesioneuroepithelioma OR esthesioneuroepitheliomas OR exostoses OR exostosis OR fibroadenoma OR fibroadenomas OR fibroadenosarcoma OR fibroadenosarcomas OR fibroadenoses OR fibroadenosis OR fibrochondrosarcoma OR fibrochondrosarcomas OR fibroelastoma OR fibroelastomas OR fibroepithelioma OR fibroepitheliomas OR fibrofolliculoma OR fibrofolliculomas OR fibroid OR fibroids OR fibrolipoma OR fibrolipomas OR fibroliposarcoma OR fibroliposarcomas OR fibroma OR fibromas OR fibromatoses OR fibromatosis OR fibromyoma OR fibromyomas OR fibromyxolipoma OR fibromyxolipomas OR fibromyxoma OR fibromyxomas OR fibroodontoma OR fibroodontomas OR fibrosarcoma OR fibrosarcomas OR fibrothecoma OR fibrothecomas OR fibroxanthoma OR fibroxanthomas OR fibroxanthosarcoma OR fibroxanthosarcomas OR ganglioblastoma OR ganglioblastomas OR gangliocytoma OR gangliocytomas OR ganglioglioma OR gangliogliomas OR ganglioneuroblastoma OR ganglioneuroblastomas OR ganglioneurofibroma OR ganglioneurofibromas OR ganglioneuroma OR ganglioneuromas OR gastrinoma OR gastrinomas OR germinoma OR germinomas OR glioblastoma OR glioblastomas OR gliofibroma OR gliofibromas OR glioma OR gliomas OR gliomatoses OR gliomatosis OR glioneuroma OR glioneuromas OR gliosarcoma OR gliosarcomas OR glomangioma OR glomangiomas OR glomangiomatoses OR glomangiomatosis OR glomangiomyoma OR glomangiomyomas OR glomangiosarcoma OR glomangiosarcomas OR glucagonoma OR glucagonomas OR gonadoblastoma OR gonadoblastomas OR gonocytoma OR gonocytomas OR granuloma OR granulomas OR granulomatoses OR granulomatosis OR gynaecomastia OR gynaecomastias OR gynandroblastoma OR gynandroblastomas OR gynecomastia OR gynecomastias OR haemangioblastoma OR haemangioblastomas OR haemangioma OR haemangiomas OR haemangiopericytoma OR haemangiopericytomas OR haemangiosarcoma OR haemangiosarcomas OR hamartoma OR hamartomas OR hemangioblastoma OR hemangioblastomas OR hemangioendothelioma OR hemangioendotheliomas OR hemangioendotheliosarcoma OR hemangioendotheliosarcomas OR hemangioma OR hemangiomas OR hemangiomatoses OR hemangiomatosis OR hemangiopericytoma OR hemangiopericytomas OR hemangioperithelioma OR hemangioperitheliomas OR hemangiosarcoma OR hemangiosarcomas OR hepatoblastoma OR hepatoblastomas OR hepatocarcinoma OR hepatocarcinomas OR hepatocholangiocarcinoma OR hepatocholangiocarcinomas OR hepatoma OR hepatomas OR hibernoma OR hibernomas OR hidradenoma OR hidradenomas OR hidrocystoma OR hidrocystomas OR histiocytoma OR histiocytomas OR hodgkin OR hodgkins OR hydatidiform OR hydradenoma OR hydradenomas OR hypernephroma OR hypernephromas OR immunocytoma OR immunocytomas OR insulinoma OR insulinomas OR kasabach-merritt OR keratoacanthoma OR keratoacanthomas OR keratoses OR keratosis OR leiomyoblastoma OR leiomyoblastomas OR leiomyofibroma OR leiomyofibromas OR leiomyoma OR leiomyomas OR leiomyomatoses OR leiomyomatosis OR leiomyosarcoma OR leiomyosarcomas OR leukaemia OR leukaemias OR leukemia OR leukemias OR leukoplakia OR leukoplakias OR Leukostases OR Leukostasis OR li fraumeni OR lipoadenoma OR lipoadenomas OR lipoblastoma OR lipoblastomas OR lipoblastomatoses OR lipoblastomatosis OR lipoma OR lipomas OR lipomatoses OR lipomatosis OR liposarcoma OR liposarcomas OR luteinoma OR luteinomas OR luteoma OR luteomas OR lymphangioendothelioma OR lymphangioendotheliomas OR lymphangioleiomyomatoses OR lymphangioleiomyomatosis OR lymphangioma OR lymphangiomas OR lymphangiomatoses OR lymphangiomatosis OR lymphangiomyoma OR lymphangiomyomas OR lymphangiomyomatoses OR lymphangiomyomatosis OR lymphangiosarcoma OR lymphangiosarcomas OR lymphoepithelioma OR lymphoepitheliomas OR lymphoma OR lymphomas OR lymphoproliferation OR lymphoproliferations OR lymphoproliferative OR macroglobulinemia OR macroglobulinemias OR macroprolactinoma OR macroprolactinomas OR malignancies OR malignancy OR malignant OR maltoma OR maltomas OR masculinovoblastoma OR masculinovoblastomas OR mastocytoma OR mastocytomas OR mastocytoses OR mastocytosis OR mcf-7 OR medulloblastoma OR medulloblastomas OR medullocytoma OR medullocytomas OR medulloepithelioma OR medulloepitheliomas OR medullomyoblastoma OR medullomyoblastomas OR melanoacanthoma OR melanoacanthomas OR melanoameloblastoma OR melanoameloblastomas OR melanocytoma OR melanocytomas OR melanoma OR melanomas OR melanomatoses OR melanomatosis OR meningioblastoma OR meningioblastomas OR meningioma OR meningiomas OR meningiomatoses OR meningiomatosis OR mesenchymoma OR mesenchymomas OR mesonephroma OR mesonephromas OR mesothelioma OR mesotheliomas OR metaplasia OR metastases OR metastasis OR metastatic OR microcarcinoma OR microcarcinomas OR microglioma OR microgliomas OR micrometastases OR micrometastasis OR mucosites OR mucositis OR myelodysplasia OR myelodysplasias OR myelodysplastic OR myelofibroses OR myelofibrosis OR myelolipoma OR myelolipomas OR myeloma OR myelomas OR myelomatoses OR myelomatosis OR myeloproliferation OR myeloproliferations OR myeloproliferative OR mycosis fungoides OR myoblastoma OR myoblastomas OR myoepithelioma OR myoepitheliomas OR myofibroblastoma OR myofibroblastomas OR myofibroma OR myofibromas OR myofibromatoses OR myofibromatosis OR myofibrosarcoma OR myofibrosarcomas OR myolipoma OR myolipomas OR myoma OR myomas OR myopericytoma OR myopericytomas OR myosarcoma OR myosarcomas OR myxofibroma OR myxofibromas OR myxolipoma OR myxolipomas OR myxoliposarcoma OR myxoliposarcomas OR myxoma OR myxomas OR neoplasia OR neoplasias OR neoplasm OR neoplasms OR neoplastic OR nephroblastoma OR nephroblastomas OR neurilemmoma OR neurilemmomas OR neurilemmomatoses OR neurilemmomatosis OR neurilemoma OR neurilemomas OR neurinoma OR neurinomas OR neuroblastoma OR neuroblastomas OR neurocytoma OR neurocytomas OR neuroepithelioma OR neuroepitheliomas OR neurofibroma OR neurofibromas OR neurofibromatoses OR neurofibromatosis OR neurofibrosarcoma OR neurofibrosarcomas OR neurolipocytoma OR neurolipocytomas OR neuroma OR neuromas OR neuronevus OR neurothekeoma OR neurothekeomas OR nevus OR nonhodgkin OR nonhodgkins OR nonseminoma OR nonseminomas OR nonseminomatous OR odontoameloblastoma OR odontoameloblastomas OR odontoma OR odontomas OR oligoastrocytoma OR oligoastrocytomas OR oligodendroglioma OR oligodendrogliomas OR oncocytoma OR oncocytomas OR oncogen OR oncogene OR oncogenes OR oncogeneses OR oncogenesis OR oncogenic OR oncogens OR oncologic OR oncologist OR oncologists OR oncology OR opsoclonus-myoclonus OR orchioblastoma OR orchioblastomas OR osteoblastoma OR osteoblastomas OR osteochondroma OR osteochondromas OR osteochondrosarcoma OR osteochondrosarcomas OR osteoclastoma OR osteoclastomas OR osteofibrosarcoma OR osteofibrosarcomas OR osteoma OR osteomas OR osteosarcoma OR osteosarcomas OR pancreatoblastoma OR pancreatoblastomas OR papilloma OR papillomas OR papillomata OR papillomatas OR papillomatoses OR papillomatosis OR parachordoma OR parachordomas OR paraganglioma OR paragangliomas OR paraneoplastic OR perineurioma OR perineuriomas OR phaeochromocytoma OR phaeochromocytomas OR pheochromoblastoma OR pheochromoblastomas OR pheochromocytoma OR pheochromocytomas OR pilomatricoma OR pilomatricomas OR pilomatrixoma OR pilomatrixomas OR pinealblastoma OR pinealblastomas OR pinealoblastoma OR pinealoblastomas OR pinealoma OR pinealomas OR pineoblastoma OR pineoblastomas OR pineocytoma OR pineocytomas OR plasmacytoma OR plasmacytomas OR pneumoblastoma OR pneumoblastomas OR pneumocytoma OR pneumocytomas OR polyembryoma OR polyembryomas OR polyhistioma OR polyhistiomas OR polyp OR polyposes OR polyposis OR polyps OR porocarcinoma OR porocarcinomas OR poroma OR poromas OR precancer OR precancerous OR precancers OR preleukaemia OR preleukaemias OR preleukemia OR preleukemias OR premalignant OR preneoplastic OR prolactinoma OR prolactinomas OR protooncogene OR protooncogenes OR pseudotumor OR pseudotumors OR pseudotumour OR pseudotumours OR reninoma OR reninomas OR reticuloendothelioma OR reticuloendotheliomas OR reticulohistiocytoma OR reticulohistiocytomas OR reticuloses OR reticulosis OR retinoblastoma OR retinoblastomas OR rhabdomyoma OR rhabdomyomas OR rhabdomyosarcoma OR rhabdomyosarcomas OR rhabdosarcoma OR rhabdosarcomas OR sarcoma OR sarcomas OR sarcomatoses OR sarcomatosis OR schwannoma OR schwannomas OR schwannomatoses OR schwannomatosis OR seminoma OR seminomas OR seminomatous OR sezary syndrome OR somatostatinoma OR somatostatinomas OR somatotropinoma OR somatotropinomas OR spermatocytoma OR spermatocytomas OR spiradenoma OR spiradenomas OR spongioblastoma OR spongioblastomas OR steatocystoma OR steatocystomas OR subependymoma OR subependymomas OR syringadenoma OR syringadenomas OR syringocystadenoma OR syringocystadenomas OR syringoma OR syringomas OR teratocarcinoma OR teratocarcinomas OR teratoma OR teratomas OR thecoma OR thecomas OR thymolipoma OR thymolipomas OR thymoma OR thymomas OR trichilemmoma OR trichilemmomas OR trichoadenoma OR trichoadenomas OR trichoblastoma OR trichoblastomas OR trichodiscoma OR trichodiscomas OR trichoepithelioma OR trichoepitheliomas OR trichofolliculoma OR trichofolliculomas OR tricholemmoma OR tricholemmomas OR tumor OR tumorgeneses OR tumorgenesis OR tumorgenic OR tumorigeneses OR tumorigenesis OR tumorigenic OR tumorogeneses OR tumorogenesis OR tumorogenic OR tumors OR tumour OR tumours OR vipoma OR vipomas OR waldenstrom OR waldenstroms OR xanthoastrocytoma OR xanthoastrocytomas OR xanthofibroma OR xanthofibromas OR xanthogranuloma OR xanthogranulomas OR xanthoma OR xanthomas OR xanthosarcoma OR xanthosarcomas).ti,ab,kw,kf | 5 815 126 |
| 4 | Pédiatrique | Exp Infant/ OR Exp Child/ OR Exp Adolescent/ OR Exp Intensive Care, Neonatal/ OR Exp Intensive Care Units, Neonatal/ OR Exp Intensive Care Units, Pediatric/ OR Exp Hospitals, Pediatric/ OR Nurses, Pediatric/ OR Exp Pediatrics/ OR Exp Pediatricians/ OR Child, Hospitalized/ OR Adolescent, Hospitalized/ OR (newborn* OR new born* OR babie* OR baby* OR infant* OR infancy OR toddler* OR preschool* OR pre school* OR child OR child' OR childs OR child's OR children* OR childhood* OR kid OR kid' OR kids OR kid's OR boy OR boy' OR boys OR boy's OR girl OR girl' OR girls OR girl's OR schoolchild* OR juvenil* OR preadolescen* OR youth* OR adolescen* OR teen OR teen' OR teens OR teen's OR teenage* OR puber OR puber' OR pubers OR puber's OR pubert* OR pubescen* OR high school* OR highschool* OR secondary school* OR paediatric* OR pediatric* OR PICU* OR neonat* OR neo nat*).ti,ab,kw,kf | 4 997 632 |
| 5 | NOT | (Exp Animals/ NOT Exp humans/) OR (guinea OR Rat OR Rats OR Mice OR Mouse OR Murine OR Animal* OR veterinar*).ti,ab,kw,kf | 6 586 625 |
| 6 | Combinaison | ((1 AND 2 AND 3 AND 4) NOT 5) AND (French OR English).lg | 10 307 |

## 3. Ovid All EBM Reviews 21 juin 2024

| 1 | Santé physique | Exp Hospitalization/ OR Exp Emergency Service, Hospital/ OR emergency medical services/ OR exp emergencies/ OR Exp Family Health/ OR health/ OR quality of life/ OR Health Status/ OR (emergenc* OR "Quality of life" OR QoL OR hrqol OR Wellbeing* OR Well being* OR Physical OR General health OR Overall health OR Poor health OR Health outcome* OR Health problem* OR Health condition* OR Health challenge* OR Health concern* OR Health issue* OR Health status OR family health OR parental health OR hospitalizat* OR hospitalisat* OR "health service use" OR "health services use" OR "healthcare use" OR "health care use" OR health service utilisation OR health services utilisation OR healthcare utilisation OR health care utilisation OR health service utilization OR health services utilization OR healthcare utilization OR health care utilization).ti,ab,kw,kf | 424 862 |
| --- | --- | --- | --- |
| 2 | Famille | Exp Family/ OR Exp caregivers/ OR (parent* OR mother* OR father* OR sibling* OR brother* OR sister* OR famil* OR caregiver* OR care-giver* OR carer OR carers OR relatives).ti,ab,kw,kf | 137 646 |
| 3 | Cancer | Exp Neoplasms/ OR Exp Medical Oncology/ OR Exp Oncology Service, Hospital/ OR Exp Oncology Nursing/ OR Exp Integrative Oncology/ OR Exp Cancer Care Facilities/ OR Exp Leukostasis/ OR Exp Myelodysplastic-Myeloproliferative diseases/ OR Exp Cancer Survivors/ OR (acanthoma OR acanthomas OR acrochordon OR acrochordons OR acrospiroma OR acrospiromas OR adamantinoma OR adamantinomas OR adenoacanthoma OR adenoacanthomas OR adenoameloblastoma OR adenoameloblastomas OR adenocanthoma OR adenocanthomas OR adenocarcinoma OR adenocarcinomas OR adenofibroma OR adenofibromas OR adenolipoma OR adenolipomas OR adenolymphoma OR adenolymphomas OR adenoma OR adenomas OR adenomatoses OR adenomatosis OR adenomatous OR adenomyoepithelioma OR adenomyoepitheliomas OR adenomyoma OR adenomyomas OR adenosarcoma OR adenosarcomas OR adenoses OR adenosis OR aesthesioneuroblastoma OR aesthesioneuroblastomas OR ameloblastoma OR ameloblastomas OR amyloidoses OR amyloidosis OR anaplasia OR anaplasias OR androblastoma OR androblastomas OR angioblastoma OR angioblastomas OR angioendothelioma OR angioendotheliomas OR angioendotheliomatoses OR angioendotheliomatosis OR angiofibroma OR angiofibromas OR angiofibrosarcoma OR angiofibrosarcomas OR angiokeratoma OR angiokeratomas OR angioleiomyoma OR angioleiomyomas OR angiolipoma OR angiolipomas OR angioma OR angiomas OR angiomatoses OR angiomatosis OR angiomyolipoma OR angiomyolipomas OR angiomyoma OR angiomyomas OR angiomyxoma OR angiomyxomas OR angioreticuloma OR angioreticulomas OR angiosarcoma OR angiosarcomas OR apudoma OR apudomas OR argentaffinoma OR argentaffinomas OR arrhenoblastoma OR arrhenoblastomas OR astroblastoma OR astroblastomas OR astrocytoma OR astrocytomas OR astroglioma OR astrogliomas OR atypia OR atypias OR baltoma OR baltomas OR basiloma OR basilomas OR Birt-Hogg-Dube OR blastoma OR blastomas OR branchioma OR branchiomas OR Buschke-Lowenstein OR cachexia OR cachexias OR cancer OR cancerous OR cancers OR carcinogen OR carcinogenesis OR carcinogenic OR carcinogens OR carcinoid OR carcinoma OR carcinomas OR carcinomatoses OR carcinomatosis OR carcinosarcoma OR carcinosarcomas OR cavernoma OR cavernomas OR cementoma OR cementomas OR cerbB2 OR ceruminoma OR ceruminomas OR chemodectoma OR chemodectomas OR cherubism OR chloroma OR chloromas OR cholangiocarcinoma OR cholangiocarcinomas OR cholangiohepatoma OR cholangiohepatomas OR cholangioma OR cholangiomas OR cholangiosarcoma OR cholangiosarcomas OR cholesteatoma OR cholesteatomas OR chondroblastoma OR chondroblastomas OR chondroma OR chondromas OR chondrosarcoma OR chondrosarcomas OR chordoma OR chordomas OR chorioadenoma OR chorioadenomas OR chorioangioma OR chorioangiomas OR choriocarcinoma OR choriocarcinomas OR chorioepithelioma OR chorioepitheliomas OR chorionepithelioma OR chorionepitheliomas OR choristoma OR choristomas OR chromaffinoma OR chromaffinomas OR cocarcinogeneses OR cocarcinogenesis OR collagenoma OR collagenomas OR comedocarcinoma OR comedocarcinomas OR condyloma OR condylomas OR corticotropinoma OR corticotropinomas OR craniopharyngioma OR craniopharyngiomas OR cylindroma OR cylindromas OR cyst OR cystadenocarcinoma OR cystadenocarcinomas OR cystadenofibroma OR cystadenofibromas OR cystadenoma OR cystadenomas OR cystoma OR cystomas OR cystosarcoma OR cystosarcomas OR cysts OR dentinoma OR dentinomas OR dermatofibroma OR dermatofibromas OR dermatofibrosarcoma OR dermatofibrosarcomas OR dermoid OR desmoid OR desmoplastic OR dictyoma OR dictyomas OR dysgerminoma OR dysgerminomas OR dyskeratoma OR dyskeratomas OR dysmyelopoieses OR dysmyelopoiesis OR dysplasia OR dysplastic OR ectomesenchymoma OR ectomesenchymomas OR elastofibroma OR elastofibromas OR enchondroma OR enchondromas OR enchondromatoses OR enchondromatosis OR endothelioma OR endotheliomas OR ependymoblastoma OR ependymoblastomas OR ependymoma OR ependymomas OR epidermoid OR epithelioma OR epitheliomas OR erythroleukaemia OR erythroleukaemias OR erythroleukemia OR erythroleukemias OR erythroplakia OR erythroplakias OR erythroplasia OR erythroplasias OR esthesioneuroblastoma OR esthesioneuroblastomas OR esthesioneuroepithelioma OR esthesioneuroepitheliomas OR exostoses OR exostosis OR fibroadenoma OR fibroadenomas OR fibroadenosarcoma OR fibroadenosarcomas OR fibroadenoses OR fibroadenosis OR fibrochondrosarcoma OR fibrochondrosarcomas OR fibroelastoma OR fibroelastomas OR fibroepithelioma OR fibroepitheliomas OR fibrofolliculoma OR fibrofolliculomas OR fibroid OR fibroids OR fibrolipoma OR fibrolipomas OR fibroliposarcoma OR fibroliposarcomas OR fibroma OR fibromas OR fibromatoses OR fibromatosis OR fibromyoma OR fibromyomas OR fibromyxolipoma OR fibromyxolipomas OR fibromyxoma OR fibromyxomas OR fibroodontoma OR fibroodontomas OR fibrosarcoma OR fibrosarcomas OR fibrothecoma OR fibrothecomas OR fibroxanthoma OR fibroxanthomas OR fibroxanthosarcoma OR fibroxanthosarcomas OR ganglioblastoma OR ganglioblastomas OR gangliocytoma OR gangliocytomas OR ganglioglioma OR gangliogliomas OR ganglioneuroblastoma OR ganglioneuroblastomas OR ganglioneurofibroma OR ganglioneurofibromas OR ganglioneuroma OR ganglioneuromas OR gastrinoma OR gastrinomas OR germinoma OR germinomas OR glioblastoma OR glioblastomas OR gliofibroma OR gliofibromas OR glioma OR gliomas OR gliomatoses OR gliomatosis OR glioneuroma OR glioneuromas OR gliosarcoma OR gliosarcomas OR glomangioma OR glomangiomas OR glomangiomatoses OR glomangiomatosis OR glomangiomyoma OR glomangiomyomas OR glomangiosarcoma OR glomangiosarcomas OR glucagonoma OR glucagonomas OR gonadoblastoma OR gonadoblastomas OR gonocytoma OR gonocytomas OR granuloma OR granulomas OR granulomatoses OR granulomatosis OR gynaecomastia OR gynaecomastias OR gynandroblastoma OR gynandroblastomas OR gynecomastia OR gynecomastias OR haemangioblastoma OR haemangioblastomas OR haemangioma OR haemangiomas OR haemangiopericytoma OR haemangiopericytomas OR haemangiosarcoma OR haemangiosarcomas OR hamartoma OR hamartomas OR hemangioblastoma OR hemangioblastomas OR hemangioendothelioma OR hemangioendotheliomas OR hemangioendotheliosarcoma OR hemangioendotheliosarcomas OR hemangioma OR hemangiomas OR hemangiomatoses OR hemangiomatosis OR hemangiopericytoma OR hemangiopericytomas OR hemangioperithelioma OR hemangioperitheliomas OR hemangiosarcoma OR hemangiosarcomas OR hepatoblastoma OR hepatoblastomas OR hepatocarcinoma OR hepatocarcinomas OR hepatocholangiocarcinoma OR hepatocholangiocarcinomas OR hepatoma OR hepatomas OR hibernoma OR hibernomas OR hidradenoma OR hidradenomas OR hidrocystoma OR hidrocystomas OR histiocytoma OR histiocytomas OR hodgkin OR hodgkins OR hydatidiform OR hydradenoma OR hydradenomas OR hypernephroma OR hypernephromas OR immunocytoma OR immunocytomas OR insulinoma OR insulinomas OR kasabach-merritt OR keratoacanthoma OR keratoacanthomas OR keratoses OR keratosis OR leiomyoblastoma OR leiomyoblastomas OR leiomyofibroma OR leiomyofibromas OR leiomyoma OR leiomyomas OR leiomyomatoses OR leiomyomatosis OR leiomyosarcoma OR leiomyosarcomas OR leukaemia OR leukaemias OR leukemia OR leukemias OR leukoplakia OR leukoplakias OR Leukostases OR Leukostasis OR li fraumeni OR lipoadenoma OR lipoadenomas OR lipoblastoma OR lipoblastomas OR lipoblastomatoses OR lipoblastomatosis OR lipoma OR lipomas OR lipomatoses OR lipomatosis OR liposarcoma OR liposarcomas OR luteinoma OR luteinomas OR luteoma OR luteomas OR lymphangioendothelioma OR lymphangioendotheliomas OR lymphangioleiomyomatoses OR lymphangioleiomyomatosis OR lymphangioma OR lymphangiomas OR lymphangiomatoses OR lymphangiomatosis OR lymphangiomyoma OR lymphangiomyomas OR lymphangiomyomatoses OR lymphangiomyomatosis OR lymphangiosarcoma OR lymphangiosarcomas OR lymphoepithelioma OR lymphoepitheliomas OR lymphoma OR lymphomas OR lymphoproliferation OR lymphoproliferations OR lymphoproliferative OR macroglobulinemia OR macroglobulinemias OR macroprolactinoma OR macroprolactinomas OR malignancies OR malignancy OR malignant OR maltoma OR maltomas OR masculinovoblastoma OR masculinovoblastomas OR mastocytoma OR mastocytomas OR mastocytoses OR mastocytosis OR mcf-7 OR medulloblastoma OR medulloblastomas OR medullocytoma OR medullocytomas OR medulloepithelioma OR medulloepitheliomas OR medullomyoblastoma OR medullomyoblastomas OR melanoacanthoma OR melanoacanthomas OR melanoameloblastoma OR melanoameloblastomas OR melanocytoma OR melanocytomas OR melanoma OR melanomas OR melanomatoses OR melanomatosis OR meningioblastoma OR meningioblastomas OR meningioma OR meningiomas OR meningiomatoses OR meningiomatosis OR mesenchymoma OR mesenchymomas OR mesonephroma OR mesonephromas OR mesothelioma OR mesotheliomas OR metaplasia OR metastases OR metastasis OR metastatic OR microcarcinoma OR microcarcinomas OR microglioma OR microgliomas OR micrometastases OR micrometastasis OR mucosites OR mucositis OR myelodysplasia OR myelodysplasias OR myelodysplastic OR myelofibroses OR myelofibrosis OR myelolipoma OR myelolipomas OR myeloma OR myelomas OR myelomatoses OR myelomatosis OR myeloproliferation OR myeloproliferations OR myeloproliferative OR mycosis fungoides OR myoblastoma OR myoblastomas OR myoepithelioma OR myoepitheliomas OR myofibroblastoma OR myofibroblastomas OR myofibroma OR myofibromas OR myofibromatoses OR myofibromatosis OR myofibrosarcoma OR myofibrosarcomas OR myolipoma OR myolipomas OR myoma OR myomas OR myopericytoma OR myopericytomas OR myosarcoma OR myosarcomas OR myxofibroma OR myxofibromas OR myxolipoma OR myxolipomas OR myxoliposarcoma OR myxoliposarcomas OR myxoma OR myxomas OR neoplasia OR neoplasias OR neoplasm OR neoplasms OR neoplastic OR nephroblastoma OR nephroblastomas OR neurilemmoma OR neurilemmomas OR neurilemmomatoses OR neurilemmomatosis OR neurilemoma OR neurilemomas OR neurinoma OR neurinomas OR neuroblastoma OR neuroblastomas OR neurocytoma OR neurocytomas OR neuroepithelioma OR neuroepitheliomas OR neurofibroma OR neurofibromas OR neurofibromatoses OR neurofibromatosis OR neurofibrosarcoma OR neurofibrosarcomas OR neurolipocytoma OR neurolipocytomas OR neuroma OR neuromas OR neuronevus OR neurothekeoma OR neurothekeomas OR nevus OR nonhodgkin OR nonhodgkins OR nonseminoma OR nonseminomas OR nonseminomatous OR odontoameloblastoma OR odontoameloblastomas OR odontoma OR odontomas OR oligoastrocytoma OR oligoastrocytomas OR oligodendroglioma OR oligodendrogliomas OR oncocytoma OR oncocytomas OR oncogen OR oncogene OR oncogenes OR oncogeneses OR oncogenesis OR oncogenic OR oncogens OR oncologic OR oncologist OR oncologists OR oncology OR opsoclonus-myoclonus OR orchioblastoma OR orchioblastomas OR osteoblastoma OR osteoblastomas OR osteochondroma OR osteochondromas OR osteochondrosarcoma OR osteochondrosarcomas OR osteoclastoma OR osteoclastomas OR osteofibrosarcoma OR osteofibrosarcomas OR osteoma OR osteomas OR osteosarcoma OR osteosarcomas OR pancreatoblastoma OR pancreatoblastomas OR papilloma OR papillomas OR papillomata OR papillomatas OR papillomatoses OR papillomatosis OR parachordoma OR parachordomas OR paraganglioma OR paragangliomas OR paraneoplastic OR perineurioma OR perineuriomas OR phaeochromocytoma OR phaeochromocytomas OR pheochromoblastoma OR pheochromoblastomas OR pheochromocytoma OR pheochromocytomas OR pilomatricoma OR pilomatricomas OR pilomatrixoma OR pilomatrixomas OR pinealblastoma OR pinealblastomas OR pinealoblastoma OR pinealoblastomas OR pinealoma OR pinealomas OR pineoblastoma OR pineoblastomas OR pineocytoma OR pineocytomas OR plasmacytoma OR plasmacytomas OR pneumoblastoma OR pneumoblastomas OR pneumocytoma OR pneumocytomas OR polyembryoma OR polyembryomas OR polyhistioma OR polyhistiomas OR polyp OR polyposes OR polyposis OR polyps OR porocarcinoma OR porocarcinomas OR poroma OR poromas OR precancer OR precancerous OR precancers OR preleukaemia OR preleukaemias OR preleukemia OR preleukemias OR premalignant OR preneoplastic OR prolactinoma OR prolactinomas OR protooncogene OR protooncogenes OR pseudotumor OR pseudotumors OR pseudotumour OR pseudotumours OR reninoma OR reninomas OR reticuloendothelioma OR reticuloendotheliomas OR reticulohistiocytoma OR reticulohistiocytomas OR reticuloses OR reticulosis OR retinoblastoma OR retinoblastomas OR rhabdomyoma OR rhabdomyomas OR rhabdomyosarcoma OR rhabdomyosarcomas OR rhabdosarcoma OR rhabdosarcomas OR sarcoma OR sarcomas OR sarcomatoses OR sarcomatosis OR schwannoma OR schwannomas OR schwannomatoses OR schwannomatosis OR seminoma OR seminomas OR seminomatous OR sezary syndrome OR somatostatinoma OR somatostatinomas OR somatotropinoma OR somatotropinomas OR spermatocytoma OR spermatocytomas OR spiradenoma OR spiradenomas OR spongioblastoma OR spongioblastomas OR steatocystoma OR steatocystomas OR subependymoma OR subependymomas OR syringadenoma OR syringadenomas OR syringocystadenoma OR syringocystadenomas OR syringoma OR syringomas OR teratocarcinoma OR teratocarcinomas OR teratoma OR teratomas OR thecoma OR thecomas OR thymolipoma OR thymolipomas OR thymoma OR thymomas OR trichilemmoma OR trichilemmomas OR trichoadenoma OR trichoadenomas OR trichoblastoma OR trichoblastomas OR trichodiscoma OR trichodiscomas OR trichoepithelioma OR trichoepitheliomas OR trichofolliculoma OR trichofolliculomas OR tricholemmoma OR tricholemmomas OR tumor OR tumorgeneses OR tumorgenesis OR tumorgenic OR tumorigeneses OR tumorigenesis OR tumorigenic OR tumorogeneses OR tumorogenesis OR tumorogenic OR tumors OR tumour OR tumours OR vipoma OR vipomas OR waldenstrom OR waldenstroms OR xanthoastrocytoma OR xanthoastrocytomas OR xanthofibroma OR xanthofibromas OR xanthogranuloma OR xanthogranulomas OR xanthoma OR xanthomas OR xanthosarcoma OR xanthosarcomas).ti,ab,kw,kf | 321 920 |
| 4 | Pédiatrique | Exp Infant/ OR Exp Child/ OR Exp Adolescent/ OR Exp Intensive Care, Neonatal/ OR Exp Intensive Care Units, Neonatal/ OR Exp Intensive Care Units, Pediatric/ OR Exp Hospitals, Pediatric/ OR Nurses, Pediatric/ OR Exp Pediatrics/ OR Exp Pediatricians/ OR Child, Hospitalized/ OR Adolescent, Hospitalized/ OR (newborn* OR new born* OR babie* OR baby* OR infant* OR infancy OR toddler* OR preschool* OR pre school* OR child OR child' OR childs OR child's OR children* OR childhood* OR kid OR kid' OR kids OR kid's OR boy OR boy' OR boys OR boy's OR girl OR girl' OR girls OR girl's OR schoolchild* OR juvenil* OR preadolescen* OR youth* OR adolescen* OR teen OR teen' OR teens OR teen's OR teenage* OR puber OR puber' OR pubers OR puber's OR pubert* OR pubescen* OR high school* OR highschool* OR secondary school* OR paediatric* OR pediatric* OR PICU* OR neonat* OR neo nat*).ti,ab,kw,kf | 372 620 |
| 5 | NOT | (Exp Animals/ NOT Exp humans/) OR (guinea OR Rat OR Rats OR Mice OR Mouse OR Murine OR Animal* OR veterinar*).ti,ab,kw,kf | 36 735 |
| 6 | Combinaison | ((1 AND 2 AND 3 AND 4) NOT 5) AND (French OR English).lg | 1 464 |

## 4. Ovid Embase 21 juin 2024

| 1 | Santé physique | Exp Hospitalization/ OR hospital emergency service/ OR emergency health service/ OR exp emergency/ OR Exp Family Health/ OR health/ OR quality of life/ OR Health Status/ OR (emergenc* OR "Quality of life" OR QoL OR hrqol OR Wellbeing* OR Well being* OR Physical OR General health OR Overall health OR Poor health OR Health outcome* OR Health problem* OR Health condition* OR Health challenge* OR Health concern* OR Health issue* OR Health status OR family health OR parental health OR hospitalizat* OR hospitalisat* OR "health service use" OR "health services use" OR "healthcare use" OR "health care use" OR health service utilisation OR health services utilisation OR healthcare utilisation OR health care utilisation OR health service utilization OR health services utilization OR healthcare utilization OR health care utilization).ti,ab,kw | 3 978 162 |
| --- | --- | --- | --- |
| 2 | Famille | Family/ OR Exp nuclear family/ OR caregiver/ OR (parent* OR mother* OR father* OR sibling* OR brother* OR sister* OR famil* OR caregiver* OR care-giver* OR carer OR carers OR relatives).ti,ab,kw | 2 799 538 |
| 3 | Cancer | Exp Neoplasm/ OR Exp Oncology/ OR cancer center/ OR Exp Oncology Nursing/ OR Leukostasis/ OR mixed myelodysplastic myeloproliferative disease/ OR Exp Cancer patien/ OR (acanthoma OR acanthomas OR acrochordon OR acrochordons OR acrospiroma OR acrospiromas OR adamantinoma OR adamantinomas OR adenoacanthoma OR adenoacanthomas OR adenoameloblastoma OR adenoameloblastomas OR adenocanthoma OR adenocanthomas OR adenocarcinoma OR adenocarcinomas OR adenofibroma OR adenofibromas OR adenolipoma OR adenolipomas OR adenolymphoma OR adenolymphomas OR adenoma OR adenomas OR adenomatoses OR adenomatosis OR adenomatous OR adenomyoepithelioma OR adenomyoepitheliomas OR adenomyoma OR adenomyomas OR adenosarcoma OR adenosarcomas OR adenoses OR adenosis OR aesthesioneuroblastoma OR aesthesioneuroblastomas OR ameloblastoma OR ameloblastomas OR amyloidoses OR amyloidosis OR anaplasia OR anaplasias OR androblastoma OR androblastomas OR angioblastoma OR angioblastomas OR angioendothelioma OR angioendotheliomas OR angioendotheliomatoses OR angioendotheliomatosis OR angiofibroma OR angiofibromas OR angiofibrosarcoma OR angiofibrosarcomas OR angiokeratoma OR angiokeratomas OR angioleiomyoma OR angioleiomyomas OR angiolipoma OR angiolipomas OR angioma OR angiomas OR angiomatoses OR angiomatosis OR angiomyolipoma OR angiomyolipomas OR angiomyoma OR angiomyomas OR angiomyxoma OR angiomyxomas OR angioreticuloma OR angioreticulomas OR angiosarcoma OR angiosarcomas OR apudoma OR apudomas OR argentaffinoma OR argentaffinomas OR arrhenoblastoma OR arrhenoblastomas OR astroblastoma OR astroblastomas OR astrocytoma OR astrocytomas OR astroglioma OR astrogliomas OR atypia OR atypias OR baltoma OR baltomas OR basiloma OR basilomas OR Birt-Hogg-Dube OR blastoma OR blastomas OR branchioma OR branchiomas OR Buschke-Lowenstein OR cachexia OR cachexias OR cancer OR cancerous OR cancers OR carcinogen OR carcinogenesis OR carcinogenic OR carcinogens OR carcinoid OR carcinoma OR carcinomas OR carcinomatoses OR carcinomatosis OR carcinosarcoma OR carcinosarcomas OR cavernoma OR cavernomas OR cementoma OR cementomas OR cerbB2 OR ceruminoma OR ceruminomas OR chemodectoma OR chemodectomas OR cherubism OR chloroma OR chloromas OR cholangiocarcinoma OR cholangiocarcinomas OR cholangiohepatoma OR cholangiohepatomas OR cholangioma OR cholangiomas OR cholangiosarcoma OR cholangiosarcomas OR cholesteatoma OR cholesteatomas OR chondroblastoma OR chondroblastomas OR chondroma OR chondromas OR chondrosarcoma OR chondrosarcomas OR chordoma OR chordomas OR chorioadenoma OR chorioadenomas OR chorioangioma OR chorioangiomas OR choriocarcinoma OR choriocarcinomas OR chorioepithelioma OR chorioepitheliomas OR chorionepithelioma OR chorionepitheliomas OR choristoma OR choristomas OR chromaffinoma OR chromaffinomas OR cocarcinogeneses OR cocarcinogenesis OR collagenoma OR collagenomas OR comedocarcinoma OR comedocarcinomas OR condyloma OR condylomas OR corticotropinoma OR corticotropinomas OR craniopharyngioma OR craniopharyngiomas OR cylindroma OR cylindromas OR cyst OR cystadenocarcinoma OR cystadenocarcinomas OR cystadenofibroma OR cystadenofibromas OR cystadenoma OR cystadenomas OR cystoma OR cystomas OR cystosarcoma OR cystosarcomas OR cysts OR dentinoma OR dentinomas OR dermatofibroma OR dermatofibromas OR dermatofibrosarcoma OR dermatofibrosarcomas OR dermoid OR desmoid OR desmoplastic OR dictyoma OR dictyomas OR dysgerminoma OR dysgerminomas OR dyskeratoma OR dyskeratomas OR dysmyelopoieses OR dysmyelopoiesis OR dysplasia OR dysplastic OR ectomesenchymoma OR ectomesenchymomas OR elastofibroma OR elastofibromas OR enchondroma OR enchondromas OR enchondromatoses OR enchondromatosis OR endothelioma OR endotheliomas OR ependymoblastoma OR ependymoblastomas OR ependymoma OR ependymomas OR epidermoid OR epithelioma OR epitheliomas OR erythroleukaemia OR erythroleukaemias OR erythroleukemia OR erythroleukemias OR erythroplakia OR erythroplakias OR erythroplasia OR erythroplasias OR esthesioneuroblastoma OR esthesioneuroblastomas OR esthesioneuroepithelioma OR esthesioneuroepitheliomas OR exostoses OR exostosis OR fibroadenoma OR fibroadenomas OR fibroadenosarcoma OR fibroadenosarcomas OR fibroadenoses OR fibroadenosis OR fibrochondrosarcoma OR fibrochondrosarcomas OR fibroelastoma OR fibroelastomas OR fibroepithelioma OR fibroepitheliomas OR fibrofolliculoma OR fibrofolliculomas OR fibroid OR fibroids OR fibrolipoma OR fibrolipomas OR fibroliposarcoma OR fibroliposarcomas OR fibroma OR fibromas OR fibromatoses OR fibromatosis OR fibromyoma OR fibromyomas OR fibromyxolipoma OR fibromyxolipomas OR fibromyxoma OR fibromyxomas OR fibroodontoma OR fibroodontomas OR fibrosarcoma OR fibrosarcomas OR fibrothecoma OR fibrothecomas OR fibroxanthoma OR fibroxanthomas OR fibroxanthosarcoma OR fibroxanthosarcomas OR ganglioblastoma OR ganglioblastomas OR gangliocytoma OR gangliocytomas OR ganglioglioma OR gangliogliomas OR ganglioneuroblastoma OR ganglioneuroblastomas OR ganglioneurofibroma OR ganglioneurofibromas OR ganglioneuroma OR ganglioneuromas OR gastrinoma OR gastrinomas OR germinoma OR germinomas OR glioblastoma OR glioblastomas OR gliofibroma OR gliofibromas OR glioma OR gliomas OR gliomatoses OR gliomatosis OR glioneuroma OR glioneuromas OR gliosarcoma OR gliosarcomas OR glomangioma OR glomangiomas OR glomangiomatoses OR glomangiomatosis OR glomangiomyoma OR glomangiomyomas OR glomangiosarcoma OR glomangiosarcomas OR glucagonoma OR glucagonomas OR gonadoblastoma OR gonadoblastomas OR gonocytoma OR gonocytomas OR granuloma OR granulomas OR granulomatoses OR granulomatosis OR gynaecomastia OR gynaecomastias OR gynandroblastoma OR gynandroblastomas OR gynecomastia OR gynecomastias OR haemangioblastoma OR haemangioblastomas OR haemangioma OR haemangiomas OR haemangiopericytoma OR haemangiopericytomas OR haemangiosarcoma OR haemangiosarcomas OR hamartoma OR hamartomas OR hemangioblastoma OR hemangioblastomas OR hemangioendothelioma OR hemangioendotheliomas OR hemangioendotheliosarcoma OR hemangioendotheliosarcomas OR hemangioma OR hemangiomas OR hemangiomatoses OR hemangiomatosis OR hemangiopericytoma OR hemangiopericytomas OR hemangioperithelioma OR hemangioperitheliomas OR hemangiosarcoma OR hemangiosarcomas OR hepatoblastoma OR hepatoblastomas OR hepatocarcinoma OR hepatocarcinomas OR hepatocholangiocarcinoma OR hepatocholangiocarcinomas OR hepatoma OR hepatomas OR hibernoma OR hibernomas OR hidradenoma OR hidradenomas OR hidrocystoma OR hidrocystomas OR histiocytoma OR histiocytomas OR hodgkin OR hodgkins OR hydatidiform OR hydradenoma OR hydradenomas OR hypernephroma OR hypernephromas OR immunocytoma OR immunocytomas OR insulinoma OR insulinomas OR kasabach-merritt OR keratoacanthoma OR keratoacanthomas OR keratoses OR keratosis OR leiomyoblastoma OR leiomyoblastomas OR leiomyofibroma OR leiomyofibromas OR leiomyoma OR leiomyomas OR leiomyomatoses OR leiomyomatosis OR leiomyosarcoma OR leiomyosarcomas OR leukaemia OR leukaemias OR leukemia OR leukemias OR leukoplakia OR leukoplakias OR Leukostases OR Leukostasis OR li fraumeni OR lipoadenoma OR lipoadenomas OR lipoblastoma OR lipoblastomas OR lipoblastomatoses OR lipoblastomatosis OR lipoma OR lipomas OR lipomatoses OR lipomatosis OR liposarcoma OR liposarcomas OR luteinoma OR luteinomas OR luteoma OR luteomas OR lymphangioendothelioma OR lymphangioendotheliomas OR lymphangioleiomyomatoses OR lymphangioleiomyomatosis OR lymphangioma OR lymphangiomas OR lymphangiomatoses OR lymphangiomatosis OR lymphangiomyoma OR lymphangiomyomas OR lymphangiomyomatoses OR lymphangiomyomatosis OR lymphangiosarcoma OR lymphangiosarcomas OR lymphoepithelioma OR lymphoepitheliomas OR lymphoma OR lymphomas OR lymphoproliferation OR lymphoproliferations OR lymphoproliferative OR macroglobulinemia OR macroglobulinemias OR macroprolactinoma OR macroprolactinomas OR malignancies OR malignancy OR malignant OR maltoma OR maltomas OR masculinovoblastoma OR masculinovoblastomas OR mastocytoma OR mastocytomas OR mastocytoses OR mastocytosis OR mcf-7 OR medulloblastoma OR medulloblastomas OR medullocytoma OR medullocytomas OR medulloepithelioma OR medulloepitheliomas OR medullomyoblastoma OR medullomyoblastomas OR melanoacanthoma OR melanoacanthomas OR melanoameloblastoma OR melanoameloblastomas OR melanocytoma OR melanocytomas OR melanoma OR melanomas OR melanomatoses OR melanomatosis OR meningioblastoma OR meningioblastomas OR meningioma OR meningiomas OR meningiomatoses OR meningiomatosis OR mesenchymoma OR mesenchymomas OR mesonephroma OR mesonephromas OR mesothelioma OR mesotheliomas OR metaplasia OR metastases OR metastasis OR metastatic OR microcarcinoma OR microcarcinomas OR microglioma OR microgliomas OR micrometastases OR micrometastasis OR mucosites OR mucositis OR myelodysplasia OR myelodysplasias OR myelodysplastic OR myelofibroses OR myelofibrosis OR myelolipoma OR myelolipomas OR myeloma OR myelomas OR myelomatoses OR myelomatosis OR myeloproliferation OR myeloproliferations OR myeloproliferative OR mycosis fungoides OR myoblastoma OR myoblastomas OR myoepithelioma OR myoepitheliomas OR myofibroblastoma OR myofibroblastomas OR myofibroma OR myofibromas OR myofibromatoses OR myofibromatosis OR myofibrosarcoma OR myofibrosarcomas OR myolipoma OR myolipomas OR myoma OR myomas OR myopericytoma OR myopericytomas OR myosarcoma OR myosarcomas OR myxofibroma OR myxofibromas OR myxolipoma OR myxolipomas OR myxoliposarcoma OR myxoliposarcomas OR myxoma OR myxomas OR neoplasia OR neoplasias OR neoplasm OR neoplasms OR neoplastic OR nephroblastoma OR nephroblastomas OR neurilemmoma OR neurilemmomas OR neurilemmomatoses OR neurilemmomatosis OR neurilemoma OR neurilemomas OR neurinoma OR neurinomas OR neuroblastoma OR neuroblastomas OR neurocytoma OR neurocytomas OR neuroepithelioma OR neuroepitheliomas OR neurofibroma OR neurofibromas OR neurofibromatoses OR neurofibromatosis OR neurofibrosarcoma OR neurofibrosarcomas OR neurolipocytoma OR neurolipocytomas OR neuroma OR neuromas OR neuronevus OR neurothekeoma OR neurothekeomas OR nevus OR nonhodgkin OR nonhodgkins OR nonseminoma OR nonseminomas OR nonseminomatous OR odontoameloblastoma OR odontoameloblastomas OR odontoma OR odontomas OR oligoastrocytoma OR oligoastrocytomas OR oligodendroglioma OR oligodendrogliomas OR oncocytoma OR oncocytomas OR oncogen OR oncogene OR oncogenes OR oncogeneses OR oncogenesis OR oncogenic OR oncogens OR oncologic OR oncologist OR oncologists OR oncology OR opsoclonus-myoclonus OR orchioblastoma OR orchioblastomas OR osteoblastoma OR osteoblastomas OR osteochondroma OR osteochondromas OR osteochondrosarcoma OR osteochondrosarcomas OR osteoclastoma OR osteoclastomas OR osteofibrosarcoma OR osteofibrosarcomas OR osteoma OR osteomas OR osteosarcoma OR osteosarcomas OR pancreatoblastoma OR pancreatoblastomas OR papilloma OR papillomas OR papillomata OR papillomatas OR papillomatoses OR papillomatosis OR parachordoma OR parachordomas OR paraganglioma OR paragangliomas OR paraneoplastic OR perineurioma OR perineuriomas OR phaeochromocytoma OR phaeochromocytomas OR pheochromoblastoma OR pheochromoblastomas OR pheochromocytoma OR pheochromocytomas OR pilomatricoma OR pilomatricomas OR pilomatrixoma OR pilomatrixomas OR pinealblastoma OR pinealblastomas OR pinealoblastoma OR pinealoblastomas OR pinealoma OR pinealomas OR pineoblastoma OR pineoblastomas OR pineocytoma OR pineocytomas OR plasmacytoma OR plasmacytomas OR pneumoblastoma OR pneumoblastomas OR pneumocytoma OR pneumocytomas OR polyembryoma OR polyembryomas OR polyhistioma OR polyhistiomas OR polyp OR polyposes OR polyposis OR polyps OR porocarcinoma OR porocarcinomas OR poroma OR poromas OR precancer OR precancerous OR precancers OR preleukaemia OR preleukaemias OR preleukemia OR preleukemias OR premalignant OR preneoplastic OR prolactinoma OR prolactinomas OR protooncogene OR protooncogenes OR pseudotumor OR pseudotumors OR pseudotumour OR pseudotumours OR reninoma OR reninomas OR reticuloendothelioma OR reticuloendotheliomas OR reticulohistiocytoma OR reticulohistiocytomas OR reticuloses OR reticulosis OR retinoblastoma OR retinoblastomas OR rhabdomyoma OR rhabdomyomas OR rhabdomyosarcoma OR rhabdomyosarcomas OR rhabdosarcoma OR rhabdosarcomas OR sarcoma OR sarcomas OR sarcomatoses OR sarcomatosis OR schwannoma OR schwannomas OR schwannomatoses OR schwannomatosis OR seminoma OR seminomas OR seminomatous OR sezary syndrome OR somatostatinoma OR somatostatinomas OR somatotropinoma OR somatotropinomas OR spermatocytoma OR spermatocytomas OR spiradenoma OR spiradenomas OR spongioblastoma OR spongioblastomas OR steatocystoma OR steatocystomas OR subependymoma OR subependymomas OR syringadenoma OR syringadenomas OR syringocystadenoma OR syringocystadenomas OR syringoma OR syringomas OR teratocarcinoma OR teratocarcinomas OR teratoma OR teratomas OR thecoma OR thecomas OR thymolipoma OR thymolipomas OR thymoma OR thymomas OR trichilemmoma OR trichilemmomas OR trichoadenoma OR trichoadenomas OR trichoblastoma OR trichoblastomas OR trichodiscoma OR trichodiscomas OR trichoepithelioma OR trichoepitheliomas OR trichofolliculoma OR trichofolliculomas OR tricholemmoma OR tricholemmomas OR tumor OR tumorgeneses OR tumorgenesis OR tumorgenic OR tumorigeneses OR tumorigenesis OR tumorigenic OR tumorogeneses OR tumorogenesis OR tumorogenic OR tumors OR tumour OR tumours OR vipoma OR vipomas OR waldenstrom OR waldenstroms OR xanthoastrocytoma OR xanthoastrocytomas OR xanthofibroma OR xanthofibromas OR xanthogranuloma OR xanthogranulomas OR xanthoma OR xanthomas OR xanthosarcoma OR xanthosarcomas).ti,ab,kw | 7 633 363 |
| 4 | Pédiatrique | Exp juvenile/ OR Exp newborn intensive care/ OR neonatal intensive care unit/ OR pediatric intensive care unit/ OR Pediatric nurse/ OR Exp Pediatrics/ OR Exp Pediatrician/ OR (newborn* OR new born* OR babie* OR baby* OR infant* OR infancy OR toddler* OR preschool* OR pre school* OR child OR child' OR childs OR child's OR children* OR childhood* OR kid OR kid' OR kids OR kid's OR boy OR boy' OR boys OR boy's OR girl OR girl' OR girls OR girl's OR schoolchild* OR juvenil* OR preadolescen* OR youth* OR adolescen* OR teen OR teen' OR teens OR teen's OR teenage* OR puber OR puber' OR pubers OR puber's OR pubert* OR pubescen* OR high school* OR highschool* OR secondary school* OR paediatric* OR pediatric* OR PICU* OR neonat* OR neo nat*).ti,ab,kw | 5 293 445 |
| 5 | NOT | (Exp Animal/ NOT Exp human/) OR conference abstract.pt. OR (guinea OR Rat OR Rats OR Mice OR Mouse OR Murine OR Animal* OR veterinar*).ti,ab,kw | 11 251 165 |
| 6 | Combinaison | ((1 AND 2 AND 3 AND 4) NOT 5) AND (French OR English).lg | 10 807 |

## 5. Clarivate Web of Science 21 juin 2024

| #1 | Santé physique | TS=(emergenc* OR "Quality of life" OR QoL OR hrqol OR Wellbeing* OR Well being* OR Physical OR General health OR Overall health OR Poor health OR Health outcome* OR Health problem* OR Health condition* OR Health challenge* OR Health concern* OR Health issue* OR Health status OR family health OR parental health OR hospitalizat* OR hospitalisat* OR health service use OR health services use OR healthcare use OR health care use OR health service utilisation OR health services utilisation OR healthcare utilisation OR health care utilisation OR health service utilization OR health services utilization OR healthcare utilization OR health care utilization) | 5 844 253 |
| --- | --- | --- | --- |
| #2 | Famille | TS=(parent* OR mother* OR father* OR sibling* OR brother* OR sister* OR famil* OR caregiver* OR "care-giver" OR carer OR carers OR relatives) | 5 172 271 |
| #3 | Cancer | TS=(acanthoma OR acanthomas OR acrochordon OR acrochordons OR acrospiroma OR acrospiromas OR adamantinoma OR adamantinomas OR adenoacanthoma OR adenoacanthomas OR adenoameloblastoma OR adenoameloblastomas OR adenocanthoma OR adenocanthomas OR adenocarcinoma OR adenocarcinomas OR adenofibroma OR adenofibromas OR adenolipoma OR adenolipomas OR adenolymphoma OR adenolymphomas OR adenoma OR adenomas OR adenomatoses OR adenomatosis OR adenomatous OR adenomyoepithelioma OR adenomyoepitheliomas OR adenomyoma OR adenomyomas OR adenosarcoma OR adenosarcomas OR adenoses OR adenosis OR aesthesioneuroblastoma OR aesthesioneuroblastomas OR ameloblastoma OR ameloblastomas OR amyloidoses OR amyloidosis OR anaplasia OR anaplasias OR androblastoma OR androblastomas OR angioblastoma OR angioblastomas OR angioendothelioma OR angioendotheliomas OR angioendotheliomatoses OR angioendotheliomatosis OR angiofibroma OR angiofibromas OR angiofibrosarcoma OR angiofibrosarcomas OR angiokeratoma OR angiokeratomas OR angioleiomyoma OR angioleiomyomas OR angiolipoma OR angiolipomas OR angioma OR angiomas OR angiomatoses OR angiomatosis OR angiomyolipoma OR angiomyolipomas OR angiomyoma OR angiomyomas OR angiomyxoma OR angiomyxomas OR angioreticuloma OR angioreticulomas OR angiosarcoma OR angiosarcomas OR apudoma OR apudomas OR argentaffinoma OR argentaffinomas OR arrhenoblastoma OR arrhenoblastomas OR astroblastoma OR astroblastomas OR astrocytoma OR astrocytomas OR astroglioma OR astrogliomas OR atypia OR atypias OR baltoma OR baltomas OR basiloma OR basilomas OR Birt-Hogg-Dube OR blastoma OR blastomas OR branchioma OR branchiomas OR Buschke-Lowenstein OR cachexia OR cachexias OR cancer OR cancerous OR cancers OR carcinogen OR carcinogenesis OR carcinogenic OR carcinogens OR carcinoid OR carcinoma OR carcinomas OR carcinomatoses OR carcinomatosis OR carcinosarcoma OR carcinosarcomas OR cavernoma OR cavernomas OR cementoma OR cementomas OR cerbB2 OR ceruminoma OR ceruminomas OR chemodectoma OR chemodectomas OR cherubism OR chloroma OR chloromas OR cholangiocarcinoma OR cholangiocarcinomas OR cholangiohepatoma OR cholangiohepatomas OR cholangioma OR cholangiomas OR cholangiosarcoma OR cholangiosarcomas OR cholesteatoma OR cholesteatomas OR chondroblastoma OR chondroblastomas OR chondroma OR chondromas OR chondrosarcoma OR chondrosarcomas OR chordoma OR chordomas OR chorioadenoma OR chorioadenomas OR chorioangioma OR chorioangiomas OR choriocarcinoma OR choriocarcinomas OR chorioepithelioma OR chorioepitheliomas OR chorionepithelioma OR chorionepitheliomas OR choristoma OR choristomas OR chromaffinoma OR chromaffinomas OR cocarcinogeneses OR cocarcinogenesis OR collagenoma OR collagenomas OR comedocarcinoma OR comedocarcinomas OR condyloma OR condylomas OR corticotropinoma OR corticotropinomas OR craniopharyngioma OR craniopharyngiomas OR cylindroma OR cylindromas OR cyst OR cystadenocarcinoma OR cystadenocarcinomas OR cystadenofibroma OR cystadenofibromas OR cystadenoma OR cystadenomas OR cystoma OR cystomas OR cystosarcoma OR cystosarcomas OR cysts OR dentinoma OR dentinomas OR dermatofibroma OR dermatofibromas OR dermatofibrosarcoma OR dermatofibrosarcomas OR dermoid OR desmoid OR desmoplastic OR dictyoma OR dictyomas OR dysgerminoma OR dysgerminomas OR dyskeratoma OR dyskeratomas OR dysmyelopoieses OR dysmyelopoiesis OR dysplasia OR dysplastic OR ectomesenchymoma OR ectomesenchymomas OR elastofibroma OR elastofibromas OR enchondroma OR enchondromas OR enchondromatoses OR enchondromatosis OR endothelioma OR endotheliomas OR ependymoblastoma OR ependymoblastomas OR ependymoma OR ependymomas OR epidermoid OR epithelioma OR epitheliomas OR erythroleukaemia OR erythroleukaemias OR erythroleukemia OR erythroleukemias OR erythroplakia OR erythroplakias OR erythroplasia OR erythroplasias OR esthesioneuroblastoma OR esthesioneuroblastomas OR esthesioneuroepithelioma OR esthesioneuroepitheliomas OR exostoses OR exostosis OR fibroadenoma OR fibroadenomas OR fibroadenosarcoma OR fibroadenosarcomas OR fibroadenoses OR fibroadenosis OR fibrochondrosarcoma OR fibrochondrosarcomas OR fibroelastoma OR fibroelastomas OR fibroepithelioma OR fibroepitheliomas OR fibrofolliculoma OR fibrofolliculomas OR fibroid OR fibroids OR fibrolipoma OR fibrolipomas OR fibroliposarcoma OR fibroliposarcomas OR fibroma OR fibromas OR fibromatoses OR fibromatosis OR fibromyoma OR fibromyomas OR fibromyxolipoma OR fibromyxolipomas OR fibromyxoma OR fibromyxomas OR fibroodontoma OR fibroodontomas OR fibrosarcoma OR fibrosarcomas OR fibrothecoma OR fibrothecomas OR fibroxanthoma OR fibroxanthomas OR fibroxanthosarcoma OR fibroxanthosarcomas OR ganglioblastoma OR ganglioblastomas OR gangliocytoma OR gangliocytomas OR ganglioglioma OR gangliogliomas OR ganglioneuroblastoma OR ganglioneuroblastomas OR ganglioneurofibroma OR ganglioneurofibromas OR ganglioneuroma OR ganglioneuromas OR gastrinoma OR gastrinomas OR germinoma OR germinomas OR glioblastoma OR glioblastomas OR gliofibroma OR gliofibromas OR glioma OR gliomas OR gliomatoses OR gliomatosis OR glioneuroma OR glioneuromas OR gliosarcoma OR gliosarcomas OR glomangioma OR glomangiomas OR glomangiomatoses OR glomangiomatosis OR glomangiomyoma OR glomangiomyomas OR glomangiosarcoma OR glomangiosarcomas OR glucagonoma OR glucagonomas OR gonadoblastoma OR gonadoblastomas OR gonocytoma OR gonocytomas OR granuloma OR granulomas OR granulomatoses OR granulomatosis OR gynaecomastia OR gynaecomastias OR gynandroblastoma OR gynandroblastomas OR gynecomastia OR gynecomastias OR haemangioblastoma OR haemangioblastomas OR haemangioma OR haemangiomas OR haemangiopericytoma OR haemangiopericytomas OR haemangiosarcoma OR haemangiosarcomas OR hamartoma OR hamartomas OR hemangioblastoma OR hemangioblastomas OR hemangioendothelioma OR hemangioendotheliomas OR hemangioendotheliosarcoma OR hemangioendotheliosarcomas OR hemangioma OR hemangiomas OR hemangiomatoses OR hemangiomatosis OR hemangiopericytoma OR hemangiopericytomas OR hemangioperithelioma OR hemangioperitheliomas OR hemangiosarcoma OR hemangiosarcomas OR hepatoblastoma OR hepatoblastomas OR hepatocarcinoma OR hepatocarcinomas OR hepatocholangiocarcinoma OR hepatocholangiocarcinomas OR hepatoma OR hepatomas OR hibernoma OR hibernomas OR hidradenoma OR hidradenomas OR hidrocystoma OR hidrocystomas OR histiocytoma OR histiocytomas OR hodgkin OR hodgkins OR hydatidiform OR hydradenoma OR hydradenomas OR hypernephroma OR hypernephromas OR immunocytoma OR immunocytomas OR insulinoma OR insulinomas OR kasabach-merritt OR keratoacanthoma OR keratoacanthomas OR keratoses OR keratosis OR leiomyoblastoma OR leiomyoblastomas OR leiomyofibroma OR leiomyofibromas OR leiomyoma OR leiomyomas OR leiomyomatoses OR leiomyomatosis OR leiomyosarcoma OR leiomyosarcomas OR leukaemia OR leukaemias OR leukemia OR leukemias OR leukoplakia OR leukoplakias OR Leukostases OR Leukostasis OR li fraumeni OR lipoadenoma OR lipoadenomas OR lipoblastoma OR lipoblastomas OR lipoblastomatoses OR lipoblastomatosis OR lipoma OR lipomas OR lipomatoses OR lipomatosis OR liposarcoma OR liposarcomas OR luteinoma OR luteinomas OR luteoma OR luteomas OR lymphangioendothelioma OR lymphangioendotheliomas OR lymphangioleiomyomatoses OR lymphangioleiomyomatosis OR lymphangioma OR lymphangiomas OR lymphangiomatoses OR lymphangiomatosis OR lymphangiomyoma OR lymphangiomyomas OR lymphangiomyomatoses OR lymphangiomyomatosis OR lymphangiosarcoma OR lymphangiosarcomas OR lymphoepithelioma OR lymphoepitheliomas OR lymphoma OR lymphomas OR lymphoproliferation OR lymphoproliferations OR lymphoproliferative OR macroglobulinemia OR macroglobulinemias OR macroprolactinoma OR macroprolactinomas OR malignancies OR malignancy OR malignant OR maltoma OR maltomas OR masculinovoblastoma OR masculinovoblastomas OR mastocytoma OR mastocytomas OR mastocytoses OR mastocytosis OR mcf-7 OR medulloblastoma OR medulloblastomas OR medullocytoma OR medullocytomas OR medulloepithelioma OR medulloepitheliomas OR medullomyoblastoma OR medullomyoblastomas OR melanoacanthoma OR melanoacanthomas OR melanoameloblastoma OR melanoameloblastomas OR melanocytoma OR melanocytomas OR melanoma OR melanomas OR melanomatoses OR melanomatosis OR meningioblastoma OR meningioblastomas OR meningioma OR meningiomas OR meningiomatoses OR meningiomatosis OR mesenchymoma OR mesenchymomas OR mesonephroma OR mesonephromas OR mesothelioma OR mesotheliomas OR metaplasia OR metastases OR metastasis OR metastatic OR microcarcinoma OR microcarcinomas OR microglioma OR microgliomas OR micrometastases OR micrometastasis OR mucosites OR mucositis OR myelodysplasia OR myelodysplasias OR myelodysplastic OR myelofibroses OR myelofibrosis OR myelolipoma OR myelolipomas OR myeloma OR myelomas OR myelomatoses OR myelomatosis OR myeloproliferation OR myeloproliferations OR myeloproliferative OR mycosis fungoides OR myoblastoma OR myoblastomas OR myoepithelioma OR myoepitheliomas OR myofibroblastoma OR myofibroblastomas OR myofibroma OR myofibromas OR myofibromatoses OR myofibromatosis OR myofibrosarcoma OR myofibrosarcomas OR myolipoma OR myolipomas OR myoma OR myomas OR myopericytoma OR myopericytomas OR myosarcoma OR myosarcomas OR myxofibroma OR myxofibromas OR myxolipoma OR myxolipomas OR myxoliposarcoma OR myxoliposarcomas OR myxoma OR myxomas OR neoplasia OR neoplasias OR neoplasm OR neoplasms OR neoplastic OR nephroblastoma OR nephroblastomas OR neurilemmoma OR neurilemmomas OR neurilemmomatoses OR neurilemmomatosis OR neurilemoma OR neurilemomas OR neurinoma OR neurinomas OR neuroblastoma OR neuroblastomas OR neurocytoma OR neurocytomas OR neuroepithelioma OR neuroepitheliomas OR neurofibroma OR neurofibromas OR neurofibromatoses OR neurofibromatosis OR neurofibrosarcoma OR neurofibrosarcomas OR neurolipocytoma OR neurolipocytomas OR neuroma OR neuromas OR neuronevus OR neurothekeoma OR neurothekeomas OR nevus OR nonhodgkin OR nonhodgkins OR nonseminoma OR nonseminomas OR nonseminomatous OR odontoameloblastoma OR odontoameloblastomas OR odontoma OR odontomas OR oligoastrocytoma OR oligoastrocytomas OR oligodendroglioma OR oligodendrogliomas OR oncocytoma OR oncocytomas OR oncogen OR oncogene OR oncogenes OR oncogeneses OR oncogenesis OR oncogenic OR oncogens OR oncologic OR oncologist OR oncologists OR oncology OR opsoclonus-myoclonus OR orchioblastoma OR orchioblastomas OR osteoblastoma OR osteoblastomas OR osteochondroma OR osteochondromas OR osteochondrosarcoma OR osteochondrosarcomas OR osteoclastoma OR osteoclastomas OR osteofibrosarcoma OR osteofibrosarcomas OR osteoma OR osteomas OR osteosarcoma OR osteosarcomas OR pancreatoblastoma OR pancreatoblastomas OR papilloma OR papillomas OR papillomata OR papillomatas OR papillomatoses OR papillomatosis OR parachordoma OR parachordomas OR paraganglioma OR paragangliomas OR paraneoplastic OR perineurioma OR perineuriomas OR phaeochromocytoma OR phaeochromocytomas OR pheochromoblastoma OR pheochromoblastomas OR pheochromocytoma OR pheochromocytomas OR pilomatricoma OR pilomatricomas OR pilomatrixoma OR pilomatrixomas OR pinealblastoma OR pinealblastomas OR pinealoblastoma OR pinealoblastomas OR pinealoma OR pinealomas OR pineoblastoma OR pineoblastomas OR pineocytoma OR pineocytomas OR plasmacytoma OR plasmacytomas OR pneumoblastoma OR pneumoblastomas OR pneumocytoma OR pneumocytomas OR polyembryoma OR polyembryomas OR polyhistioma OR polyhistiomas OR polyp OR polyposes OR polyposis OR polyps OR porocarcinoma OR porocarcinomas OR poroma OR poromas OR precancer OR precancerous OR precancers OR preleukaemia OR preleukaemias OR preleukemia OR preleukemias OR premalignant OR preneoplastic OR prolactinoma OR prolactinomas OR protooncogene OR protooncogenes OR pseudotumor OR pseudotumors OR pseudotumour OR pseudotumours OR reninoma OR reninomas OR reticuloendothelioma OR reticuloendotheliomas OR reticulohistiocytoma OR reticulohistiocytomas OR reticuloses OR reticulosis OR retinoblastoma OR retinoblastomas OR rhabdomyoma OR rhabdomyomas OR rhabdomyosarcoma OR rhabdomyosarcomas OR rhabdosarcoma OR rhabdosarcomas OR sarcoma OR sarcomas OR sarcomatoses OR sarcomatosis OR schwannoma OR schwannomas OR schwannomatoses OR schwannomatosis OR seminoma OR seminomas OR seminomatous OR sezary syndrome OR somatostatinoma OR somatostatinomas OR somatotropinoma OR somatotropinomas OR spermatocytoma OR spermatocytomas OR spiradenoma OR spiradenomas OR spongioblastoma OR spongioblastomas OR steatocystoma OR steatocystomas OR subependymoma OR subependymomas OR syringadenoma OR syringadenomas OR syringocystadenoma OR syringocystadenomas OR syringoma OR syringomas OR teratocarcinoma OR teratocarcinomas OR teratoma OR teratomas OR thecoma OR thecomas OR thymolipoma OR thymolipomas OR thymoma OR thymomas OR trichilemmoma OR trichilemmomas OR trichoadenoma OR trichoadenomas OR trichoblastoma OR trichoblastomas OR trichodiscoma OR trichodiscomas OR trichoepithelioma OR trichoepitheliomas OR trichofolliculoma OR trichofolliculomas OR tricholemmoma OR tricholemmomas OR tumor OR tumorgeneses OR tumorgenesis OR tumorgenic OR tumorigeneses OR tumorigenesis OR tumorigenic OR tumorogeneses OR tumorogenesis OR tumorogenic OR tumors OR tumour OR tumours OR vipoma OR vipomas OR waldenstrom OR waldenstroms OR xanthoastrocytoma OR xanthoastrocytomas OR xanthofibroma OR xanthofibromas OR xanthogranuloma OR xanthogranulomas OR xanthoma OR xanthomas OR xanthosarcoma OR xanthosarcomas) | 6 533 580 |
| #4 | Pédiatrique | TS=(newborn* OR new born* OR babie* OR baby* OR infant* OR infancy OR toddler* OR preschool* OR pre school* OR child OR child' OR childs OR child's OR children* OR childhood* OR kid OR kid' OR kids OR kid's OR boy OR boy' OR boys OR boy's OR girl OR girl' OR girls OR girl's OR schoolchild* OR juvenil* OR preadolescen* OR youth* OR adolescen* OR teen OR teen' OR teens OR teen's OR teenage* OR puber OR puber' OR pubers OR puber's OR pubert* OR pubescen* OR high school* OR highschool* OR secondary school* OR paediatric* OR pediatric* OR PICU* OR neonat* OR neo nat*) | 4 180 578 |
| #5 | NOT | TS=(guinea OR Rat OR Rats OR Mice OR Mouse OR Murine OR Animal* OR veterinar*) | 5 197 060 |
| #6 | Combinaison | ((#1 AND #2 AND #3 AND #4) NOT #5)  Refined By:Languages: English or French | 18 149 |

**eTable 2. Completed data extraction table**

See excel file.

**eTable 3. Results reported by outcomes**

See excel file.

**eTable 4. Risk of bias assessment (JBI Checklist for Prevalence Studies)**

See excel file.

**References**

1. Slama T, Mader L, Zarkovic M, et al. Chronic health conditions after childhood Langerhans cell histiocytosis: Results from the Swiss Childhood Cancer Survivor Study. *J Cancer Surviv*. Feb 14 2024;doi:10.1007/s11764-024-01544-z

2. Streefkerk N, Teepen JC, Feijen EAM, et al. The cumulative burden of self-reported, clinically relevant outcomes in long-term childhood cancer survivors and implications for survivorship care: A DCCSS LATER study. *Cancer*. Apr 15 2024;130(8):1349-1358. doi:10.1002/cncr.35148

3. Auger N, Marcoux S, Begin P, et al. Matched cohort study of hospitalization in children who have siblings with cancer. *Cancer*. Apr 15 2022;128(8):1684-1691. doi:10.1002/cncr.34115

4. Baker KS, Ness KK, Weisdorf D, et al. Late effects in survivors of acute leukemia treated with hematopoietic cell transplantation: a report from the Bone Marrow Transplant Survivor Study. *Leukemia*. Dec 2010;24(12):2039-47. doi:10.1038/leu.2010.210

5. Ehrhardt MJ, Chen Y, Sandlund JT, et al. Late Health Outcomes After Contemporary Lymphome Malin de Burkitt Therapy for Mature B-Cell Non-Hodgkin Lymphoma: A Report From the Childhood Cancer Survivor Study. *J Clin Oncol*. Oct 1 2019;37(28):2556-2570. doi:10.1200/JCO.19.00525

6. Essig S, Li Q, Chen Y, et al. Risk of late effects of treatment in children newly diagnosed with standard-risk acute lymphoblastic leukaemia: a report from the Childhood Cancer Survivor Study cohort. *Lancet Oncol*. Jul 2014;15(8):841-51. doi:10.1016/S1470-2045(14)70265-7

7. Rueegg CS, Michel G, Wengenroth L, et al. Physical performance limitations in adolescent and adult survivors of childhood cancer and their siblings. *PLoS One*. 2012;7(10):e47944. doi:10.1371/journal.pone.0047944

8. Dixon SB, Chen Y, Yasui Y, et al. Reduced Morbidity and Mortality in Survivors of Childhood Acute Lymphoblastic Leukemia: A Report From the Childhood Cancer Survivor Study. *Journal of clinical oncology : official journal of the American Society of Clinical Oncology*. Oct 10 2020;38(29):3418-3429. doi:10.1200/JCO.20.00493

9. Hayek S, Gibson TM, Leisenring WM, et al. Prevalence and Predictors of Frailty in Childhood Cancer Survivors and Siblings: A Report From the Childhood Cancer Survivor Study. *J Clin Oncol*. Jan 20 2020;38(3):232-247. doi:10.1200/JCO.19.01226

10. Bouwman E, Penson A, de Valk M, et al. Unhealthy lifestyle behaviors, overweight, and obesity among childhood cancer survivors in the Netherlands: A DCCSS LATER study. *Cancer*. Aug 15 2024;130(16):2856-2872. doi:10.1002/cncr.35338

11. Tacyildiz N, Cakmak HM, Unal E, et al. Evaluation of late effects during a 21-year follow-up of pediatric Hodgkin lymphoma survivors: Experience of a pediatric cancer center in Turkey, as a developing country model. *Indian J Cancer*. Apr 1 2024;61(2):282-289. doi:10.4103/ijc.IJC_912_20

12. Lu Q, Krull KR, Leisenring W, et al. Pain in long-term adult survivors of childhood cancers and their siblings: A report from the Childhood Cancer Survivor Study. *Pain*. 2011;152(11):2616-2624. doi:10.1016/j.pain.2011.08.006

13. Mottonen M, Uhari M. Stomach ache and headache among the siblings of children with acute lymphoblastic leukaemia. *Acta Paediatr*. Sep 1995;84(9):1072-3. doi:10.1111/j.1651-2227.1995.tb13828.x

14. Penson A, Walraven I, Bronkhorst E, et al. Chronic fatigue in childhood cancer survivors is associated with lifestyle and psychosocial factors; a DCCSS LATER study. *ESMO Open*. Dec 2023;8(6):102044. doi:10.1016/j.esmoop.2023.102044

15. van der Plas E, Darji H, Srivastava DK, et al. Risk factors for neurocognitive impairment, emotional distress, and poor quality of life in survivors of pediatric rhabdomyosarcoma: A report from the Childhood Cancer Survivor Study. *Cancer*. Jun 15 2024;130(12):2224-2236. doi:10.1002/cncr.35236

16. Claessens JJM, Penson A, Bronkhorst EM, et al. Reproductive outcomes and reproductive health care utilization among male survivors of childhood cancer: A DCCSS-LATER study. *Cancer*. Mar 15 2024;130(6):995-1004. doi:10.1002/cncr.35119

17. National Center for Health Statistics. Health, United States: Key Statistics from the National Survey of Family Growth - I Listing. Hyattsville, Maryland. 2022. Accessed October, 2024.

18. Byrne J, Fears TR, Whitney C, Parry DM. Survival after retinoblastoma: long-term consequences and family history of cancer. *Med Pediatr Oncol*. Mar 1995;24(3):160-5. doi:10.1002/mpo.2950240304

19. Infante-Rivard C, Amre DK. Congenital anomalies in children with acute lymphoblastic leukaemia and in their family. *Int J Epidemiol*. Apr 2001;30(2):350-2. doi:10.1093/ije/30.2.350

20. Oeffinger KC, Mertens AC, Sklar CA, et al. Chronic health conditions in adult survivors of childhood cancer. *N Engl J Med*. Oct 12 2006;355(15):1572-82. doi:10.1056/NEJMsa060185

21. Sherief LM, Beshir MR, Salem GM, et al. Intrafamilial Transmission of Hepatitis C Virus Among Families of Infected Pediatric Oncology Patients. *Pediatr Infect Dis J*. Jul 2019;38(7):692-697. doi:10.1097/INF.0000000000002299

22. Molgaard-Hansen L, Glosli H, Jahnukainen K, et al. Quality of health in survivors of childhood acute myeloid leukemia treated with chemotherapy only: a NOPHO-AML study. *Pediatr Blood Cancer*. Dec 15 2011;57(7):1222-9. doi:10.1002/pbc.22931

23. Ng AK, Li S, Recklitis C, et al. A comparison between long-term survivors of Hodgkin's disease and their siblings on fatigue level and factors predicting for increased fatigue. *Ann Oncol*. Dec 2005;16(12):1949-55. doi:10.1093/annonc/mdi407

24. Buchbinder D, Oeffinger K, Franco-Villalobos C, et al. Tobacco Use Among Siblings of Childhood Cancer Survivors: A Report From the Childhood Cancer Survivor Study. *Pediatr Blood Cancer*. Feb 2016;63(2):326-33. doi:10.1002/pbc.25719

25. Lown EA, Mertens AC, Korcha RA, et al. Prevalence and predictors of risky and heavy alcohol consumption among adult siblings of childhood cancer survivors. *Psychooncology*. May 2013;22(5):1134-43. doi:10.1002/pon.3121
